# Mechanisms of *Salmonella* Attachment and Survival on In-Shell Black Peppercorns, Almonds, and Hazelnuts

**DOI:** 10.3389/fmicb.2020.582202

**Published:** 2020-10-23

**Authors:** Ye Li, Joelle K. Salazar, Yingshu He, Prerak Desai, Steffen Porwollik, Weiping Chu, Palma-Salgado Sindy Paola, Mary Lou Tortorello, Oscar Juarez, Hao Feng, Michael McClelland, Wei Zhang

**Affiliations:** ^1^Department of Food Science and Nutrition, Illinois Institute of Technology, Bedford Park, IL, United States; ^2^Division of Food Processing Science and Technology, U.S. Food and Drug Administration, Bedford Park, IL, United States; ^3^Department of Microbiology and Molecular Genetics, University of California, Irvine, Irvine, CA, United States; ^4^Department of Food Science and Human Nutrition, University of Illinois at Urbana-Champaign, Urbana, IL, United States; ^5^Department of Biology, Illinois Institute of Technology, Chicago, IL, United States

**Keywords:** *Salmonella*, low-moisture food, transposon sequencing, interstrain difference, storage condition, population dynamics, confocal laser scanning microscopy, gene function analysis

## Abstract

*Salmonella enterica* subspecies I (ssp 1) is the leading cause of hospitalizations and deaths due to known bacterial foodborne pathogens in the United States and is frequently implicated in foodborne disease outbreaks associated with spices and nuts. However, the underlying mechanisms of this association have not been fully elucidated. In this study, we evaluated the influence of storage temperature (4 or 25°C), relative humidity (20 or 60%), and food surface characteristics on the attachment and survival of five individual strains representing *S. enterica* ssp 1 serovars Typhimurium, Montevideo, Braenderup, Mbandaka, and Enteritidis on raw in-shell black peppercorns, almonds, and hazelnuts. We observed a direct correlation between the food surface roughness and *S. enterica* ssp 1 attachment, and detected significant inter-strain difference in survival on the shell surface under various storage conditions. A combination of low relative humidity (20%) and ambient storage temperature (25°C) resulted in the most significant reduction of *S. enterica* on shell surfaces (*p* < 0.05). To identify genes potentially associated with *S. enterica* attachment and survival on shell surfaces, we inoculated a library of 120,000 random transposon insertion mutants of an *S.* Enteritidis strain on almond shells, and screened for mutant survival after 1, 3, 7, and 14 days of storage at 20% relative humidity and 25°C. Mutants in 155 *S*. Enteritidis genes which are involved in carbohydrate metabolic pathways, aerobic and anaerobic respiration, inner membrane transport, and glutamine synthesis displayed significant selection on almond shells (*p* < 0.05). Findings of this study suggest that various food attributes, environmental factors, and an unexpectedly complex metabolic and regulatory network in *S. enterica* ssp 1 collectively contribute to the bacterial attachment and survival on low moisture shell surface, providing new data for the future development of knowledge-based intervention strategies.

## Introduction

*Salmonella enterica* subspecies I serovars have been implicated in a number of multi-state foodborne disease outbreaks associated with nuts and spices in recent decades in the United States. In 2004, serovar Enteritidis was linked to raw almonds in an outbreak in 12 states, leading to 29 cases of infections (Centers for Disease Control Prevention [CDC], 2004). In 2010, 272 cases of illnesses were reported in an outbreak of *S.* Montevideo linked to black pepper (Centers for Disease Control Prevention [CDC], 2010). In 2016, pistachios were implicated in an outbreak of *S.* Montevideo and *S.* Senftenberg infections, which sickened 11 persons in 9 states (Centers for Disease Control Prevention [CDC], 2018). Other reported salmonellosis outbreaks linked to nuts and nut-associated products included Turkish pine nuts contaminated by *S.* Enteritidis, nut butter by *S.* Braenderup, and peanut butter by *S.* Typhimurium and *S.* Mbandaka (Scheil et al., 1998; Centers for Disease Control Prevention [CDC], 2009, 2011, 2014). These foodborne outbreaks highlight the need for a thorough examination of the mechanisms of *S. enterica* survival and persistence on nuts and spices.

*Salmonella enterica* may contaminate raw nuts and spices at any stage of the food production process, including growing, harvesting, handling, processing, packaging, and storage. Following harvest, nuts and spices may be directly sold as ready-to-eat food without further heat treatment such as roasting; thus, *S. enterica* potentially residing on the surfaces may not be eradicated prior to packaging and retail. Storage temperature and relative humidity (RH) are considered to be two important environmental factors that influence *S. enterica* survival on nut and spice surfaces. Harvested raw nuts are usually stored at an RH between 55 and 60% to ensure quality and to limit mold growth (Codex Alimentarius, 2014). Ambient storage (25°C) is often used to sustain flavor quality but may lead to shortened shelf life of raw nuts, compared to storage at 4°C (Senesi et al., 1996; Bruhn et al., 2010).

Once attached to the shells, *S. enterica* is capable of surviving for long periods of time on low water activity foods, including nuts. For instance, Beuchat and Heaton (1975) demonstrated that *S. enterica* survive more than 32 weeks at low temperatures (≤ 5°C) on pecans. Blessington et al. (2012) reported that *S. enterica* survive under ambient storage conditions (23–25°C, 25–35% RH) on walnuts for more than 97 days. Extended survival of *S. enterica* was also documented by Kimber et al. (2012) on pistachios for at least 12 months at various storage temperatures including −19, 4, and 24°C (Centers for Disease Control Prevention [CDC], 2009).

Despite many previous efforts to assess the extended survival period of *S. enterica* on various raw nuts, scientific data are scarce on how different food surface characteristics, such as surface roughness, may impact bacterial attachment and survival, and how various *S. enterica* serovars and strains may differ in their relative ability to attach to and survive on nut shell surface. In addition, the molecular basis underlying the ability of *S. enterica* to survive on raw in-shell nuts remains largely unknown.

In this study, we assessed the population dynamics of several foodborne outbreak-associated *S. enterica* serovars on one spice, i.e., black peppercorns, and two different raw nuts, i.e., almonds and hazelnuts, under different storage conditions. We further employed electron microscopy and high-throughput sequencing techniques to explore the physical and molecular mechanisms that *S. enterica* uses to attach to and survive on the surface of these foods. Findings of this study shed new light on the behavior and physiology of this pathogen on nuts and peppercorns, furthering a better understanding of its dissemination and transmission that results in human foodborne illnesses.

## Materials and Methods

### Bacterial Strains

Five bacterial strains representing *S. enterica* subspecies I serovars Typhimurium (strain ATCC 19585), Montevideo (strain FDA691341), Braenderup (strain ATCC BAA-1739), Mbandaka (strain FDA660151), and Enteritidis (strain ATCC 13076) were used in this study (Table 1). Single colonies of each *S. enterica* stock culture were recovered in Brain Heart Infusion (BHI, Becton Dickinson and Co., Franklin Lakes, NJ) broth at 37°C with shaking at 200 rpm for 24 h. A total of 100 μl cultures were spread onto the surface of BHI agar plates and incubated for 24 h at 37°C. Bacterial cells were then harvested from the BHI agar plates using a spreader and 1 mL of pre-warmed (37°C) phosphate buffered saline (PBS, pH 7.4), spun down, and re-suspended in 1 mL of PBS. Bacterial suspensions were transferred to 15 mL sterile centrifuge tubes and adjusted to a final concentration of 10–11 log CFU/mL with PBS prior to inoculation. The initial inoculum level was determined by serial dilution and enumeration on Xylose Lysine Deoxycholate (XLD, Becton Dickinson and Co.) agar plates.

**TABLE 1:** *S. enterica* strains used in this study.

**Serovar**	**Strain ID**	**Source and description of use**
Enteritidis	ATCC 13076	American Type Culture Collection (ATCC), Manassas, VA
Enteritidis	P125109	Sequenced isolate, from Wellcome Sanger Institute, Hinxton, United Kingdom; for transposon-mutagenesis library construction
Typhimurium	ATCC 19585	From American Type Culture Collection (ATCC), Manassas, VA
Braenderup	ATCC BAA-1739	From American Type Culture Collection (ATCC), Manassas, VA
Montevideo	FDA 691341	Isolated from tahini (Lebanon), provided by US FDA
Mbandaka	FDA 660151	Isolated from whole sesame seed (Canada), provided by US FDA

### Food Samples

Whole black peppercorns, raw in-shell almonds, and raw in-shell hazelnuts were selected for this study to assess the attachment and survival of *S. enterica*. Peppercorn, almond, and hazelnut samples were purchased from local vendors and stored in sterile 500 mL containers at room temperature prior to experimental procedures. The background microbiota on the un-inoculated samples was determined to be below 1 log CFU/g prior to our experiments, and remained largely unchanged throughout the 2-week storage after inoculation with *S. enterica*.

### Microbiological Assessment of *S. enterica* Population Dynamics on Peppercorns, Almonds, and Hazelnuts

Approximately 20 g of each food was surface-inoculated by mixing each sample with 1 mL of 10–11 log CFU/mL of individual cultures of *S.* Typhimurium, *S.* Montevideo, *S.* Braenderup, *S.* Mbandaka, and *S.* Enteritidis in 4 × 8 in whirl-pak bags (Nasco, Fort Atkinson, WI). The bags were then hand-massaged for 10 min. Inoculated samples were dried for 2 h in a biosafety cabinet and then placed in incubators under ambient conditions (i.e., 25°C and 60% RH) to simulate typical commercial storage and packaging conditions. Saturated salt solutions (Supplementary Table 1) were used to maintain the RH for storage as previously reported (Greenspan, 1977). At 0, 1, 3, 7, and 14 days, inoculated samples were transferred to 50 mL sterile centrifuge tubes containing 20 mL PBS for enumeration of viable *S. enterica*.

We developed a vortex-based separation method to collect *S. enterica* cells from the surface of artificially inoculated nut samples. At each sampling time point, 20 g of each sample was submerged in 20 mL PBS buffer in a 50 mL conical polypropylene centrifuge tube and vortexed at 300 rpm for 10 min. The bacterial cell suspension was collected. An additional 20 mL PBS buffer was then added to the sample in the same tube. A subsequent vortex at 3000 rpm for 10 min with 3 mm diameter sterile glass beads was performed to remove the remaining surface-attached *S. enterica* from the shells. The bacterial cell suspension from the subsequent 3000 rpm vortex was collected and mixed with that from the previous vortex at 300 rpm for enumeration. This method allowed maximum removal of surface-attached bacterial cells from shell samples while minimizing potential mechanical cell lysis. Two different culture media, BHI agar and XLD agar, were used to recover bacterial cells and to differentiate healthy versus impaired *S. enterica* cells. *S. enterica* was enumerated by serial dilution of the homogenates with PBS, followed by plating onto both of these agars.

An additional storage temperature at 4°C was tested to compare the attachment and survival behaviors of *S*. Enteritidis and *S*. Typhimurium on black peppercorn, almond, and hazelnut surfaces to those at 25°C under 20 or 60% relative humidity conditions, respectively.

### Measurement of Shell Surface Roughness Using CLSM

The surface roughness of black peppercorns, almond shells, and hazelnut shells was measured using a confocal laser scanning microscopy (CLSM)-based method developed by Fransisca and Feng (2012). Briefly, each shell sample was firmly attached to a microscope slide in twelve replicates. CLSM (Nanofocus, Surf explorer^®^) was used to capture three-dimensional (3D) images of the surfaces at 20 × magnification with a measurement area of 800 μm × 800 μm. In order to obtain high-quality 3D images, we adjusted width and height based on the size of the samples. Three random spots were measured for each sample. Images were analyzed using the MountainsMap software (Digital Surf, Besançon, France) to convert 3D surface texture and surface morphological traits to ISO 25178-2 3D parameters (ISO, 2012). The surface area roughness parameter Sq is calculated as follows:

$$S\,q=\sqrt{\frac{1}{A}\,\int \int_{A}Z^{2}\,\left(x,y\right)\,dx\,dy}$$

where *Z*^2^ (*x, y*) represents the squared distance from individual heights and depths to the mean plane.

### Imaging of *S. enterica* Surface Attachment on Shell Surface Using SEM

Images were captured using scanning electron microscopy (SEM) to illustrate the patterns and morphology of *S. enterica* during initial attachment to the surface of the samples. Samples were prepared using the methods developed by Wen et al. (2009) and He et al. (2013), with minor modifications. Briefly, shell samples were dissected into small pieces (∼1 cm^2^) and integral and flat pieces were selected for imaging. Entire shell surfaces were spot-inoculated with 10 μL of 10–11 log CFU/mL of individual cultures of *S.* Enteritidis and *S.* Typhimurium and then air-dried for 24 h in a biosafety cabinet at ambient temperature (25°C). Inoculated shell samples were fixed with a primary fixative (2.5% glutaraldehyde in 0.1M cacodylate buffer, pH 7.2) for 2 h followed by a secondary fixative (1% osmium tetroxide aqueous solution in 0.1M cacodylate buffer, pH 7.2) without light for 1 h at room temperature. Fixed samples were dehydrated by sequential washes with electron microscopy-grade ethanol solutions (25, 50, 75, 85, 95, and 100%), and then dried using 100% hexamethyldisilazane (HMDS). Chemical reagents were purchased from Electron Microscopy Sciences (EMS) (Hatfield, PA). Samples were mounted on aluminum studs, sputter coated with Au and Pt to an 8 nm thickness, and examined using a JSM-6320F field emission scanning electron microscope (JEOL USA Inc, Peabody, MA) at an instrument magnification of 10,000 × at the University of Illinois Chicago microscopy core facility.

### Transposon Sequencing Analysis of *S. enterica* Serovar Enteritidis PT4 on Almonds

A transposon insertion library in a fully sequenced *S. enterica* serovar Enteritidis PT4 strain was utilized, which had been constructed as previously described (de Moraes et al., 2017, 2018; Jayeola et al., 2020). Briefly, the EZ-Tn5 < T7/KAN-2 > promoter insertion kit (Epicentre Technologies Corporation, Madison, WI) was employed, and specific primers were used to add N_18_ random barcodes to both sides of the EZ-Tn5 transposon for sequencing analysis (Supplementary Table 2). The final transposon insertion library consisted of approximately 120,000 random insertion mutants.

To identify specific genes that were involved in *S. enterica* survival on almond shells, we adopted a published method (de Moraes et al., 2017; Jayeola et al., 2020), with minor modifications. Briefly, the entire Tn5 mutant library was surface-inoculated on in-shell almonds using the inoculation protocol described above. After 0, 1, 3, 7, and 14 days of storage under ambient conditions, *S. enterica* cells were collected from almond shells as described above, with minor modifications. Specifically, homogenates collected from the two-step separation were enriched in Luria Bertani (LB; Becton Dickinson and Co.) broth with 50 μg/mL kanamycin at 37°C with shaking for 8 h to reach ∼8 log CFU/mL. Enriched *S. enterica* cells were pelleted and plated onto LB agar containing kanamycin and incubated at 37°C for 24 h. Genomic DNA was extracted using Generation Capture Columns from Qiagen (Hilden, Germany). The DNA was subsequently used as template for PCR using primers flanking the N_18_ barcode, and the frequency of each barcode was determined by Illumina sequencing, as previously described (de Moraes et al., 2017, 2018; George et al., 2018; Jayeola et al., 2020).

### Mapping and Identification of *S. enterica* Mutants Under Selection

To map each transposon insertion site on the genome, custom Perl scripts were used to trim the Tn5 priming sites and the corresponding N_18_ barcodes from the raw sequence reads. Bowtie2 (Version 2.3.0) was used to align the filtered reads to the *S.* Enteritidis P125109 reference genome. Custom Perl scripts were used to identify each mapped read to its corresponding N_18_ barcode.

For use in fitness experiments, the overall abundance of the N_18_ barcodes in each annotated gene was summed in each strand separately, thereby reporting genome features on both strands of each feature; e.g., coding and non-coding strands. Aggregated abundances for the input and output libraries were analyzed for statistical significance with EdgeR (Version 3.5), to identify mutants under positive or negative selection (Robinson et al., 2009; Robinson and McCarthy, 2010).

### Statistical Analysis

All experiments were repeated at least three times, each with additional technical duplicates and independent cultures, to ensure data reproducibility for statistical analysis. *S. enterica* counts collected on selective XLD agar plates were used to minimize false positive counts due to background microflora. *D*-values were determined with the Bigelow model (Edwards and Berry, 1987). The data were also analyzed using *post hoc* Tukey’s honest significant difference (HSD) test, student’s *t*-test (GraphPad Prism software package, Version 5; Microsoft Excel), and analyses of variance (ANOVA), where a *p*-value of less than 0.05 was considered significant.

## Results

### *S. enterica* Inter-Strain Variation in Attachment and Survival on Nuts and Spice

Among hundreds of different pathogenic *S. enterica* ssp 1 serovars that can cause salmonellosis, only a handful have been implicated in foodborne disease outbreaks associated with raw nuts and spices. We therefore compared the relative ability of different *S. enterica* ssp 1 serovars to attach to and survive on raw nuts and spices, which serves as an initial step of food contamination. Five *S. enterica* strains, representing ssp 1 serovars Typhimurium, Montevideo, Braenderup, Mbandaka, and Enteritidis, and three shell surface, black peppercorns, almonds, and hazelnuts, were assessed.

Immediately after inoculation and air-drying, and prior to storage (i.e., at Day 0), we collected and enumerated *S. enterica* cells attached on black peppercorns, almonds and hazelnuts to evaluate the initial level of bacterial attachment for each strain on each type of shell surface. All five *S. enterica* strains displayed better initial attachment to black peppercorns and almonds than to hazelnuts; however, significant differences were observed among different strains (Supplementary Table 3). For example, *S*. Mbandaka displayed the highest initial attachment levels, ranging from 7.42 to 8.18 log CFU/g. In contrast, the attachment of *S*. Enteritidis on hazelnuts and black pepper was significantly lower than that of all other tested strains on the same surfaces (*p* < 0.05), suggesting *S. enterica* strains do differ in their capability to attach to nut or spice surfaces.

**FIGURE 1:**
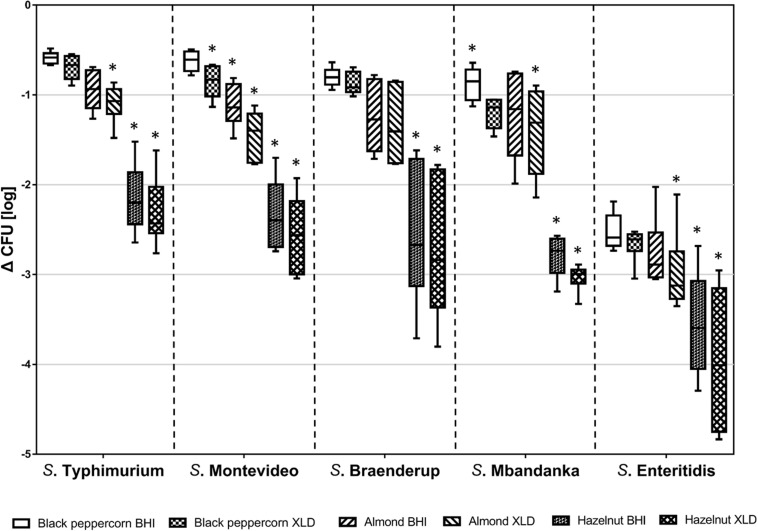
Log reduction of five *S. enterica* strains on shell surfaces over 14-day storage at 25°C and 60% relative humidity. The box-and-whiskers represent the maximum, 75th percentile, median (center line), 25th percentile, and the minimum values from top to bottom of the box, respectively. * indicates significant log reduction of *S. enterica* of the same strain (*p* < 0.05) among different shell surfaces. Error bars indicate standard deviations of means (*n* = 6).

Over the course of the 14-day storage period at 25°C and 60% RH, all five *S. enterica* strains displayed an overall population reduction on different shell surfaces (Figure 1 and Supplementary Table 3). However, the rates of reduction were different among *S. enterica* strains and surfaces (*p* < 0.05). For instance, the *S.* Typhimurium population decreased by less than 1 log CFU/g on black pepper and almonds, whereas the *S.* Enteritidis population declined rapidly by 3 to 4 log CFU/g on hazelnuts. *S*. Montevideo, *S*. Braenderup, and *S.* Mbandaka showed similar behaviors of survival to that of *S*. Typhimurium.

Counting of surviving CFUs was performed on both a rich medium (BHI) and a more stringent selective medium (XLD). CFUs obtained on XLD were lower, suggesting that some bacterial cells were impaired due to the stresses that *S. enterica* encountered on the shells. Background microbiota including bacteria other than *Salmonella* and molds contributed to the higher microbial counts on non-selective BHI. To minimize the false positive plate counts of *S. enterica* due to the background microbiota, we chose to use XLD counts for further data analysis.

To better illustrate the relative ability of the bacterial strains to survive under the same storage conditions (i.e., 25°C and 60% RH), we calculated the *D*-values (i.e., time required to reach a 90% population reduction) of different *S. enterica* strains on the three types of surfaces based on XLD plate counts (Table 2). The majority of tested *S. enterica* strains survived better on black pepper and almond, with *D*-values ranging from 12 to 22 days, than on hazelnut, with *D*-values ranging from 4 to 7 days. Again, *S*. Enteritidis was substantially less capable of surviving on all three types of surfaces, with *D*-values that were 2–3 times lower than those of the other *S. enterica* strains.

**TABLE 2:** *D* values for different *S. enterica* strains on the surface of nut samples stored at 25°C and 60% relative humidity.

**Food sample**	**Mean ± SD *D* value(r^2^) (days)**				
	***S.* Typhimurium**	***S.* Montevideo**	***S.* Braenderup**	***S.* Mbandaka**	***S.* Enteritidis**
Black peppercorn	22.6 ± 3.3 (0.90)^Aa^	18.7 ± 4.6 (0.91)^Aa^	17.8 ± 2.0 (0.90)^Aa^	12.9 ± 1.9 (0.97)^Ab^	6.1 ± 0.6 (0.84)^Ac^
Almond	15.7 ± 4.7 (0.72)^Ba^	13.0 ± 3.3 (0.79)^Ba^	14.0 ± 7.1 (0.83)^Aa^	13.1 ± 4.8 (0.89)^Aa^	5.3 ± 0.9 (0.89)^Ab^
Hazelnut	6.6 ± 1.4 (0.93)^Ca^	5.8 ± 0.6 (0.97)^Cab^	7.1 ± 1.9 (0.91)^Babc^	4.9 ± 0.2 (0.99)^Bbc^	4.0 ± 1.0 (0.90)^Bc^

*D values (time required to reach 90% population reduction) were calculated based on three independent biological replicates recovered on XLD agar plates. Different capital letters indicate significant difference (*p* < 0.05) among *D* values of the same strain on the surface of different food samples (columns); different lowercase letters indicate significant difference (*p* < 0.05) among *D* values of different strains on the surface of the same food sample (rows). *r*^2^, coefficients of determination.*

### Effects of Storage Temperature and Relative Humidity on *S. enterica* Survival on Nuts

**FIGURE 2:**
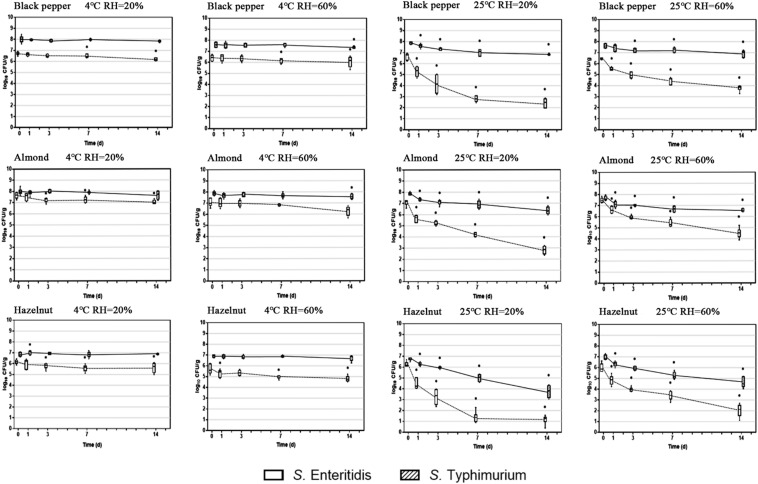
Population dynamics of *S. enterica* Enteritidis and Typhimurium on black peppercorn, almond, and hazelnut over 14 days of storage. Two storage temperatures (4 and 25°C) and two relative humidity conditions (20 and 60%) were evaluated. The solid lines represent *S.* Typhimurium and the dotted lines represent *S.* Enteritidis. The box-and-whiskers represent the maximum, 75th percentile, median (center line), 25th percentile, and the minimum values from top to bottom of the box, respectively. * Indicates significant population changes of the same *S. enterica* strain (*p* < 0.05) in comparison to initial inoculation. Error bars indicate standard deviations of means (*n* = 6).

Two important environmental factors that have been recognized to have a collective impact on *S. enterica* survival on food surfaces are storage temperature and relative humidity. In order to evaluate the effect of these factors on *S. enterica* survival on peppercorns, almonds, and hazelnuts we chose to compare two serovars, *S.* Enteritidis and *S.* Typhimurium, which appeared to be substantially different from each other with regards to surface attachment and survival under ambient storage condition, using three additional storage conditions. Figure 2 illustrates the population dynamics of these two strains on the surface of black peppercorn, almond and hazelnut under 4°C/20% RH, 4°C/60% RH, 25°C/20% RH, and 25°C/60% RH, respectively, over a 14-day storage.

At 4°C, bacterial populations of both *S*. Enteritidis and *S*. Typhimurium did not show a significant reduction on all three types of surfaces, independent from RH. However, at 25°C, both *S. enterica* strains displayed a population decrease on the three surfaces. For instance, *S*. Enteritidis showed a reduction of ∼ 5 log CFU/g on hazelnut at 20% RH. Interestingly, ambient storage temperature seemed to be less impactful on *S*. Typhimurium survival on the surfaces, with an average population reduction of < 2 log CFU/g. Our results show that a combination of ambient temperature (25°C) and low humidity (20%) led to an overall higher bacterial reduction for *S. enterica* on peppercorns, almonds, and hazelnuts. Furthermore, storage temperature appeared to have a more direct impact on overall *S. enterica* survival than relative humidity.

### Effects of Surface Topology on *S. enterica* Attachment

**FIGURE 3:**
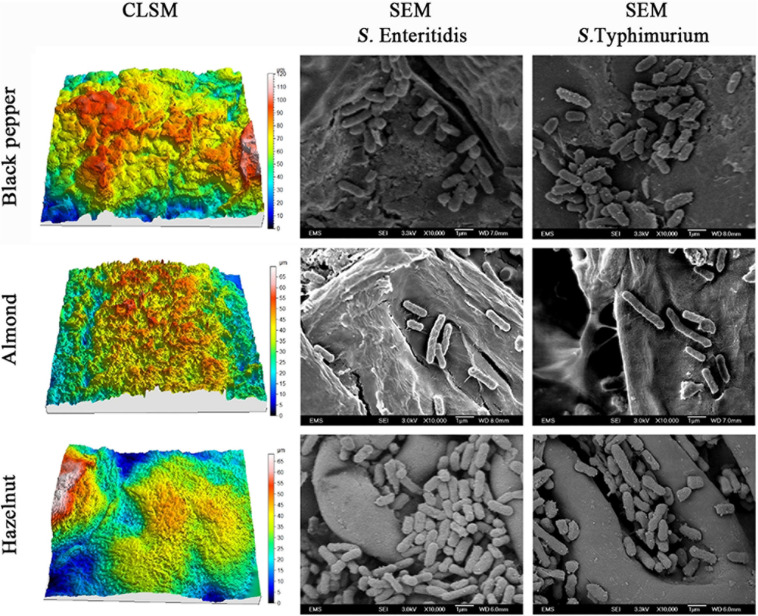
Reconstructed three-dimensional CLSM images showing the surface topology of black peppercorn, almond, and hazelnut at 1,000 × magnifications, and SEM images showing the locations of surface-attached *S. enterica* Enteritidis and Typhimurium cells on different shell surfaces at 10,000 × magnification.

To explore the physical mechanisms of *S. enterica* attachment on raw nut shells and peppercorns, we compared the patterns and morphology of attached bacteria in relation to the roughness of the different surfaces using scanning microscopy. The left column in Figure 3 shows the reconstructed 3-D confocal laser scanning microscopy (CLSM) images of the surfaces of black peppercorns, almonds and hazelnuts. Surface roughness was calculated based on the 3-D images and accompanying areal roughness parameters by Matlab as described (Wang et al., 2009; Fransisca and Feng, 2012). Hazelnuts had a relatively smooth surface with an Sq value of 4.8 ± 1.1 μm, whereas black peppercorns had a rough surface with an Sq value of 20.4 ± 3.2 μm. The numbers of attached *S. enterica* cells were positively correlated with the surface roughness for all five *S. enterica* strains on all tested surfaces when stored at 25°C and 60% RH. The right column in Figure 3 shows the scanning electron microscopy (SEM) imaging of surface-attached *S. enterica* cells, and demonstrated that *S. enterica* tended to aggregate in the uneven spaces of the surfaces, such as grooves and cavities, with minor morphological changes.

### *S.* Enteritidis Genes Under Selection on the Surface of Almonds

**FIGURE 4:**
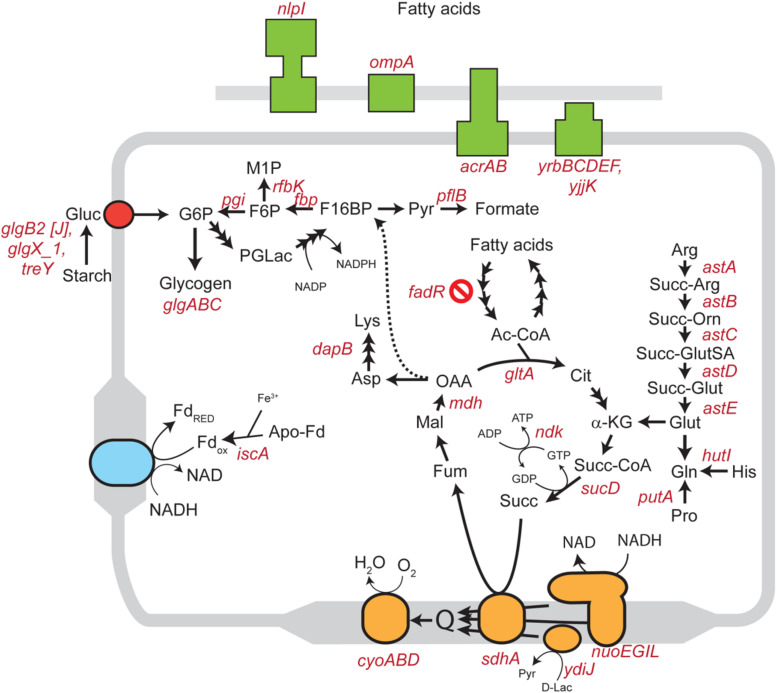
Schematic diagram showing various *S. enterica* genes involved in carbohydrate, lipid, amino acid, and energy metabolism for which mutants displayed significantly negative selection on almond surfaces after 7-day storage at 25°C. Genes involved in aerobic and anaerobic respiration, cholesterol transport systems, and inner membrane transport systems in *S.* Enteritidis are highlighted in orange, blue, green and red colors, respectively. Abbreviations: Gluc, glucose; G6P, glucose 6-phosphate; F6P, fructose 6-phosphate; M1P, D-mannitol 1-phosphate; F16BP, fructose 1,6-bisphosphate; Pyr, pyruvate; PGLac, phosphogluconolactone; Fd, ferredoxin; Ac-CoA, acetyl coenzyme A; Cit, citrate; α-KG, alpha-ketoglutarate; Succ-CoA, succinyl-CoA; Succ, succinate; Fum, fumarate; Mal, malate; OAA, oxaloacetate; Asp, aspartic acid; Lys, lysine; Arg, arginine; Succ-Arg, N_2_-succinylarginine; Succ-Orn, N_2_-succinylornithine; Succ-GluSA, N-succinyl-L-glutamate 5-semialdehyde; Succ-Glut, N-succinyl-L-glutamate; Glut, glutamate; Gln, glutamine; His, histidine; Pro, proline; D-Lac, D-lactate.

We inoculated a random transposon insertion library of an *S.* Enteritidis strain on almond shells to screen for *S. enterica* genes that were either positively or negatively selected during bacterial residence on the shell surface over a 7-day ambient storage (25°C under 60% RH). We monitored the proportion of each transposon mutant at days 0, 1, 3, 7, and 14 (Supplementary Table 6). Using this approach, we identified a total of 102 negatively selected and 53 positively selected *S. enterica* genes with statistical significance (Supplementary Tables 4, 5), including genes encoding metabolic regulators, pathogenicity island proteins, membrane proteins, fimbrial subunits, transcriptional regulators, and hypothetical proteins. Table 3 lists a subset of *S. enterica* genes that were under strong selection on almond shells with functional annotations. More than half of the negatively selected genes were involved in various metabolism pathways. Figure 4 illustrates a number of negatively selected genes involved in carbohydrate metabolic pathways, aerobic and anaerobic respiration, inner membrane transport, and glutamine synthesis. In addition, we found that every successfully assayed mutant in the pSEN virulence plasmid was positively selected. As the plasmid has its own origin of replication, this observation was likely due to the copy number of the plasmid being higher relative to the copy number of the genome at later time points.

**TABLE 3:** Selected *S. enterica* Enteritidis genes under selection on almonds at 14 days of storage at 25°C and 60% relative humidity (*p* < 0.05).

**Functional category**	**Gene**	**Mutant selection**	**Annotation**
Virulence	*sspH2*	+	Leucine-rich repeat-containing protein secreted by SPI-2 and induced by the SPI-2 regulator *ssrA/B*
Metabolism	*glgP*	+	Glycogen phosphorylase involved in glycogen metabolism
	*gltA*	−	Type II citrate synthase functions in TCA cycle, glyoxylate cycle and respiration
	*sdhA*	−	Succinate dehydrogenase flavoprotein subunit, together with *sdhB* exhibited succinate dehydrogenase activity in the absence of *sdhC/D* which are the membrane components and form cytochrome b556
	*cyoA*	−	Cytochrome o ubiquinol oxidase subunit II involved in aerobic electron transport
	*fbp*	−	Fructose-1,6-bisphosphatase catalyzes the formation of D-fructose-6-phosphate from fructose-1,6-bisphosphate
	*astA*	−	Arginine succinyltransferase involved in L-arginine degradation via AST pathway
Cell adhesion and surface structure	*yiaF*	+	Putative outer membrane lipoprotein
	*ompA*	−	Outer membrane protein A precursor
	*yhdA*	+	Putative lipoprotein
	*yfgC*	−	Putative inner membrane or exported
	*lpfE*	+	Long polar fimbrial minor protein; PFE protein precursor
Transport	*yrbD*	−	Putative transport protein involved in a phospholipid transport pathway
	*ftsE*	+	Putative ATP-binding protein of an ATP-binding cassette transporter
	*acrA*	−	Acridine efflux pump
	*yrbK*	+	Lipopolysaccharide export system protein
DNA replication and stress response	*dnaK*	−	Molecular chaperone; heat shock protein 70
DNA replication and stress response	*hslU*	−	ATP-dependent protease ATP-binding subunit; heat shock protein involved in degradation of misfolded proteins
	*hslV*	−	ATP-dependent protease peptidase subunit; heat shock protein involved in degradation of misfolded proteins
	*pspA*	−	Phage shock protein involved in maintaining membrane potential under membrane stress conditions
	*pspC*	+	DNA-binding transcriptional activator activated the phage-shock-protein operon in response to phage infection, exposure to ethanol or osmotic shock
	*phoP*	+	DNA-binding transcriptional regulator involved in magnesium starvation and stress
Cell cycle	*nlpI*	−	Lipoprotein involved in cell division
Transcription	*fruR*	−	DNA-binding transcriptional regulator
	*purR [D]*	−	Putative LacI family transcriptional regulator
	*glpR*	−	DNA-binding transcriptional repressor; glycerol-3-phosphate regulon repressor
	*zur*	−	Zinc uptake transcriptional repressor acts as a negative controlling element, employing Zn(2 +) as a cofactor to bind the operator of the repressed genes *znuACB*
	*cytR*	+	DNA-binding transcriptional regulator
Unknown function	*smpA*	−	Hypothetical small protein A
	*yebA*	+	Hypothetical protein
	*yibP*	+	Hypothetical protein

Interestingly, a large number of Tn5 mutants in *S*. Enteritidis which were strongly selected on almonds were also found to be strongly selected in *S. enterica* on pistachios (Jayeola et al., 2020). Table 4 lists the *S. enterica* genes that were selected on both almonds and pistachios. Supplementary Table 7 provides a complete list of genes that were either consistently or differentially selected on almonds and pistachios. Among the genes for which mutants were positively selected in both *S.* Enteritidis on almonds and *S*. Typhimurium on pistachios was the *glgP* gene. Strikingly, mutants in *glgP* were selected in the opposite direction than in *glgABC* encoded in the same operon. We selected this gene for further analysis. We constructed a deletion mutant of the *glgP* gene in *S.* Enteritidis and a complemented strain containing *glgP* encoded on a plasmid. The constructs displayed no significant difference in growth in BHI at 37°C for 48 h when compared to the wild-type strain (data not shown), suggesting that this gene is not needed in rich media. After 7-days storage on almond shells at 25°C and 60% RH, *S.* Enteritidis Δ*glgP* displayed better survival than its complemented strain and the wild-type strain (data not shown). To our knowledge, no positively selected mutant in *Salmonella* has previously been confirmed using a single gene deletion mutant on a food product.

**TABLE 4:** Selected *S. enterica* genes under selection on both almonds and pistachios (*p* < 0.05).

**Functional category**	**Gene**	**Mutant selection**	**Annotation**
Metabolism	*fbp*	−	Fructose-1,6-bisphosphatase catalyzes the formation of D-fructose-6-phosphate from fructose-1,6-bisphosphate
	*glgA*	−	Glycogen synthase; ADP-glucose transglucosylase
	*glgB*	−	1,4-alpha-glucan (glycogen) branching enzyme, GH-13-type
	*glgC*	−	Glucose-1-phosphate adenylyltransferase
	*glgB2 [J]*	−	Putative alpha amylase; malto-oligosyltrehalose trehalohydrolase
	*treY*	−	Putative glycosyl hydrolase; malto-oligosyltrehalose synthase
	*glgX_1*	−	Putative glycosyl hydrolase; putative glycogen debranching protein
	*astD*	−	Succinylglutamic semialdehyde dehydrogenase
	*cyoD*	−	Cytochrome o ubiquinol oxidase subunit IV
	*pflB*	−	Pyruvate formate acetyltransferase 1
	*rbsK*	−	Ribokinase
	*rfaL*	−	O-antigen ligase; oligosaccharide repeat unit polymerase
	*rfaK*	−	Putative hexose transferase; lipopolysaccharide 1,2-N-acetylglucosaminetransferase; lipopolysaccharide core biosynthetic protein
	*rfaJ*	−	Lipopolysaccharide glucosyltransferase; UDP-D-glucose/galactosyl; lipopolysaccharide 1,2-glucosyltransferase
	*rfaI*	−	Lipopolysaccharide 1,3-galactosyltransferase
	*rfbP*	−	Undecaprenol-phosphate galactosephosphotransferase/O-antigen transferase
	*rfbK*	−	Phosphomannomutase
	*rfbN*	−	Rhamnosyl transferase
	*rfbC*	−	dTDP-4,deoxyrhamnose 3,5 epimerase; dTDP-4-dehydrorhamnose 3,5-epimerase
	*rfbD*	−	dTDP-4-dehydrorhamnose reductase
	*rfbB*	−	dTDP-glucose 4,6 dehydratase; NAD(P) binding
	*Pnp*	−	Polyribonucleotide nucleotidyltransferase; polyadenylation bacterial, bacterial RNA-metabolizing Zn-dependent hydrolases
	*pgi*	−	Phosphoglucose isomerase
	*barA*	−	Hybrid sensory histidine kinase; signal transduction histidine-protein kinase
	*ubiB_2*	−	FMN reductase; NAD(P)H-flavin reductase
	*glgP*	+	Glycogen phosphorylase involved in glycogen metabolism
	*treF*	+	Cytoplasmic trehalase
Cell adhesion and surface structure	*ompA*	−	Outer membrane protein A precursor
	*ygaU*	−	Putative LysM domain
	*hflC*	−	FtsH protease regulator
	*yhhL*	+	Putative membrane protein
	*yiaF*	+	Putative outer membrane lipoprotein
	*yhdA*	+	Putative lipoprotein
	*dsbA*	+	Periplasmic protein disulfide isomerase I; thiol-disulfide interchange protein DabA precursor
Metal binding	*iscA*	−	Putative iron-sulfur cluster assembly protein
	*cafA*	−	Ribonuclease G
Cell cycle	*nlpI*	−	Lipoprotein involved in cell division
	*ftsL*	+	Membrane bound cell division protein
	*ftsN*	+	Essential cell division protein
	*ytfB*	+	Putative cell envelope opacity-associated protein A
DNA replication and stress response	*hslU*	−	ATP-dependent protease ATP-binding subunit; heat shock protein involved in degradation of misfolded proteins
	*hslV*	−	ATP-dependent protease peptidase subunit; heat shock protein involved in degradation of misfolded proteins
	*proQ*	−	Putative solute/DNA competence effector; RNA chaperone; activator of ProP
	*uvrA*	−	Excinuclease ABC subunit A
	*uvrB*	−	Excinuclease ABC subunit B
	*uvrC*	−	Excinuclease ABC subunit C
	*uvrY*	−	Response regulator
	*topA*	−	DNA topoisomerase I; omega protein I
	*ydaA*	−	Putative universal stress protein
	*cspC*	−	Cold shock-like protein
	*hscA*	−	Chaperone protein
	*phoP*	+	DNA-binding transcriptional regulator involved in magnesium starvation and stress
	*stpA*	+	DNAbinding protein, nucleoid-associated
Transcription	*hupA*	−	Transcriptional regulator HU subunit alpha; histone-like DNA-binding protein
	*hupB*	−	Transcriptional regulator; DNA-binding protein
	*zur*	−	Zinc uptake transcriptional repressor acts as a negative controlling element, employing Zn(2 +) as a cofactor to bind the operator of the repressed genes *znuACB*
	*rpoS*	−	RNA polymerase sigma factor
	*trpR*	−	Trp operon repressor
	*hha*	−	Hemolysin expression-modulating protein
	*yegW*	−	Putative gntR-family regulatory protein
	*hfq*	−	RNA-binding protein; host factor-I protein(HF-I)
	*rnb*	−	Exoribonuclease II involved in mRNA degradation
	*yhbJ*	−	Putative kinase; contains putative P-loop; RNase adapter protein
	*arcZ [P]*	−	Post-transcriptional regulator represses sdaC, STM3216 and tpx mRNAs
Translation	*miaA*	−	tRNA delta(2)-isopentenylpyrophosphate transferase; IPP transferase; isopentenyltransferase
	*thiI*	−	Thiamine biosynthesis protein
	*clpA*	+	ATP-dependent Clp protease; ATP-binding specificity subunit of the ClpA-ClpP
	*fusA*	+	Translation elongation factor G; EF-G;
Unknown function	*yibP*	+	Hypothetical protein

*Data on pistachios are from Jayeola et al. (2020).*

## Discussion

A mechanistic understanding of the attachment and survival of *S. enterica* on nuts and spices is an important step toward developing effective control and mitigation strategies to reduce or eliminate pathogen contamination in these food products. Yet, this aspect of food safety has not been thoroughly elucidated by previous studies. Our study demonstrated that different *S. enterica* strains display variable abilities to attach to and survive on raw shell surfaces. For instance, *S*. Enteritidis was less capable of attaching to nut shells, whereas *S*. Mbandaka showed a better capability of attachment. In addition, the overall rate of survival of different *S. enterica* strains attached to shell surface also appeared to be quite variable, suggesting strain- or serovar-specific behavior should be taken into consideration– an important aspect when evaluating and validating a particular mitigation strategy on raw in-shell foods.

It is known that desiccation can inhibit *S. enterica* proliferation and metabolic functions (Deng et al., 2012). Water activity (a_*w*_) on raw shells can range from 0.321 ± 0.20 on peanuts (Fong and Wang, 2016) to 0.585 ± 0.003 on chia seeds. On shell surfaces, *S. enterica* displayed an overall population reduction over the 14-day period at ambient storage conditions. This reduction was likely caused by the limited water availability for bacterial use (Uesugi et al., 2006; Keller et al., 2012; Blessington et al., 2013; Bowman et al., 2015). We observed that the magnitude of bacterial reduction varied among different *S. enterica* spp 1 strains. In addition to inhibiting bacterial growth, desiccation can also trigger various stress response mechanisms, enabling the pathogen to persist in low moisture environments for even longer periods of time. Some published studies have shown that once adapted to a desiccated environment, *S. enterica* can survive for months or even years in orchards and surrounding environments (Beuchat and Heaton, 1975; Blessington et al., 2012; Kimber et al., 2012). A consistent finding of these studies was that the populations of *S. enterica* initially declined quickly under low moisture stress, followed by a slower population decline and long term persistence (Gurtler et al., 2014), similar to what we observed in this study.

Storage temperature plays a critical role in *S. enterica* survival on shell surface. In this study, we demonstrated that the rate of reduction of *S. enterica* stored at ambient temperature (25°C) was significantly higher than that under refrigeration temperature (4°C). Note that the rate of the population reduction of *S.* Enteritidis on almond shells under ambient storage conditions (0.21 ± 0.03 log CFU/g per day) was higher than the rates observed for *S. enterica* on other nut products under similar conditions. These conditions included almond kernels (0.25 ± 0.05 log CFU/g per month), pecan halves (0.15 log CFU/g per month), and peanut kernels (0.22 log CFU/g per month) (Uesugi et al., 2006; Brar et al., 2015; Santillana Farakos et al., 2016). Refrigeration temperature, in contrast, was protective for *S. enterica* on nut shells, consistent with other studies (Uesugi et al., 2006; Brar et al., 2015).

One factor, among possibly many others, which can directly influence the attachment and survival of *S. enterica* on nuts and peppercorns, is the shell surface roughness (Ra). Our study showed that all tested *S. enterica* strains survived better on the rougher surface of almond shells and black peppercorns than on the smoother surface of hazelnut shells. A positive correlation between surface roughness and survival of *S. enterica* on surfaces was observed when samples were stored at 25°C and 60% RH (Figure 1 and Supplementary Table 3). This is consistent with studies of other bacterial pathogens in other food models. For instance, Wang et al. (2009) reported that *E. coli* O157:H7 displayed a higher attachment rate on the rough cantaloupe surface (Ra = 14.18 ± 0.25 μm) than on a smooth apple surface (Ra = 1.43 ± 0.13 μm). Our SEM analysis further demonstrated that *S. enterica* tended to aggregate at locations of uneven surface structures, such as grooves and cavities (Figure 3), which could provide a “safe harbor” for the pathogen to attach and escape decontamination.

A number of previous studies suggested that the overall bacterial attachment and survival on various biotic and abiotic surfaces can be mediated by genes encoding transcription factors, regulators, virulence determinants, and cell surface components such as cellulose, flagella, and fimbriae (Barak et al., 2005; Marvasi et al., 2013; Salazar et al., 2013; Tan et al., 2016; de Moraes et al., 2017; Jayeola et al., 2020). We used a library of over 120,000 mutant strains, each with a random Tn5 transposon insertion, to identify the subset of mutants that is under selection during up to 14 days of ambient storage on almond shells. When a Tn5 mutant is lost or less abundant in an “output” pool collected after an experimental treatment, compared to an “input” pool prior to treatment (initial Tn5 library inoculated on almond shells), this “negative selection” suggests a loss of bacterial fitness in a particular environment. This fitness loss indicates an important functional role for the location of the transposon insertion in bacterial survival. Our transposon sequencing analysis identified a few hundred genes in *S*. Enteritidis from a wide variety of functional groups for which mutants were under selection after 7-day storage on almond shells under ambient conditions (Table 3). We identified a number of *S. enterica* genes related to the bacterial stress response, DNA repair systems, and cellular metabolisms, for which mutants were negatively selected on almond shells. These included genes involved in carbohydrate metabolic pathways, aerobic and anaerobic respiration, inner membrane transport, and glutamine synthesis (Figure 4).

Two prominent genes that were negatively selected in our screen are *dnaK* and *pspA*. The chaperone protein DnaK was previously found to play a role in *S.* Enteritidis survival in low moisture peanut oil (Deng et al., 2012). In *E. coli*, this protein can interact with the co-chaperone DnaJ under different stress conditions, thereby preventing the aggregation of stress-denatured proteins (Liberek et al., 1991). Gene *pspA* encodes the phage shock protein A (PspA) in the *pspABCDE* operon, which helps to maintain proton-motive force (ΔμH +) of the cell under membrane stress conditions (Kleerebezem et al., 1996). PspA expression is also induced under more general environmental stress conditions, including heat (50°C), ethanol (10%) or hyperosmolarity shock (Model et al., 1997).

Other negatively selected *S. enterica* genes on almonds included *sdhA* and the *ast* operon. Gene *sdhA* encodes a membrane-bound subunit of the succinate dehydrogenase (SDH) flavoprotein in the tricarboxylic acid (TCA) cycle (Park et al., 1995). The *astABCDE* operon encodes enzymes in the ammonia-producing arginine succinyltransferase (AST) pathway and controls arginine catabolism during bacterial aerobic exponential growth (Schneider et al., 1998). The negative selection of stress response, DNA repair and metabolism-related genes mentioned above in *S*. Enteritidis suggested a collective contribution of various pathways in promoting and sustaining *S. enterica* survival and persistence on almond shells.

We found that a number of genes belonging to cell adhesion and surface structures were under positive selection when mutated by Tn5, suggesting that expression of these genes is disadvantageous for *S. enterica* survival on almond shells. Notably, mutations in *phoP* and *glgP* were positively selected in both *S*. Enteritidis on almonds and in *S*. Typhimurium on pistachios. PhoP is part of a two-component system that controls virulence, Mg^2+^ homeostasis, and resistance to antimicrobial peptides, and controls 9% of genes in *Salmonella* (Soncini et al., 1996; Walters et al., 2006; Baker et al., 2014; Colgan et al., 2016; Bansal et al., 2017). Gene *glgP* encodes glycogen phosphorylase, which catalyzes the rate-limiting step in glycogen breakdown by removing glucose units from the polysaccharide (Alonso-Casajus et al., 2006). In *E. coli*, a close relative of *S. enterica*, the ability to metabolize glycogen is associated with a rapid response to carbon source starvation (Sekar et al., 2020). That glycogen catabolism is disadvantageous in two different *Salmonella* serovars in two different nuts is surprising, and warrants additional experimental validation. Another example is a gene encoding the fimbrial adhesin protein LpfE, which was under positive selection. LpfE is part of the *lpfABCC’DE* fimbrial operon, which encodes long polar fimbriae – a structure that contributes to the attachment of *S.* Typhimurium to murine small intestine Peyer’s patch cells and participates in the adherence phenotype during EHEC O157:H7 microcolony formation (Baumler et al., 1996; Torres et al., 2002). The *lpfABCC’DE* fimbrial operon in EHEC O113:H21 has also been shown to increase bacterial adherence to the epithelial cells of Chinese hamster ovary-K1 (CHO-K1) cells (Doughty et al., 2002). The positive selection of *lpfE* on almonds suggested that expression of this gene is, in contrast, disadvantageous for *S. enterica* attachment to and/or survival on the almond surface.

Interestingly, a comparison of our *S.* Enteritidis genes under selection on almonds with genes under selection from a recent study using *S.* Typhimurium on pistachios (Jayeola et al., 2020), revealed that a majority of these genes were commonly selected on both (Table 4). For example, genes *hslU*, *fbp*, and *zur* were all negatively selected on both almonds and pistachios. Gene *hslU* encodes an ATP-dependent protease for the degradation of misfolded polypeptides during heat-shock (Rohrwild et al., 1996; Burton et al., 2005). Gene *fbp* encodes a key enzyme, fructose-1,6-bisphosphatase, which functions in the Calvin cycle and the gluconeogenesis pathway (Nel and Terblanche, 1992; Daie, 1993). This gene is also related to bacterial attachment and biofilm formation in *Streptococcus* infections (Christie et al., 2002; Barros et al., 2015; Fessia et al., 2019). Zinc uptake transcriptional repressor Zur acts as a negative controlling element when employing Zn^2+^ as a cofactor to bind the operator of the repressed genes cluster *znuACB* (Patzer and Hantke, 2000). The strong negative selection of these genes collectively indicated the involvement of their corresponding proteins in the survival of *S. enterica* on low moisture nut shells.

Some limitations inherent to the nature of our experiments did exist. For instance, we used artificial surface inoculation to create very high bacterial loads on nut shells, a typical approach used in bacterial survival and challenge studies. Such high level of bacterial loads may be unrealistic in the event of natural contamination of nuts and spices. In addition, the relative ability of *S. enterica* attachment and survival on different shell surface was evaluated by separating bacterial cells from the shells at specific sampling time points and enumerating on BHI and XLD media. *S. enterica* cells that became severely injured and non-culturable but remained infectious could be underestimated. Several other factors such as bacterial acclimatization during air-drying and recovery can also influence the initial bacterial loads on shell surfaces. Therefore, the initial inoculation levels may not accurately reflect the bacterial ability of attachment and survival on respective shell surfaces, which remains a major technical challenge for this type of studies.

Future studies on the population dynamics of *S. enterica* over a longer storage period (e.g., 10–40 weeks or more) will provide additional information on how this pathogen behaves on nuts and spices. Use of lower inoculation levels may better reflect contamination events that are likely to occur in foodborne disease outbreaks. While in this study we demonstrated striking concordance in the *S. enterica* genes under selection in two different serovars on two different nuts (Table 4), studies on other low-moisture foods, using mutant libraries of other *S. enterica* strains and serovars, will provide further evidence for genes and pathways which are critical for the survival and persistence of this pathogen. Additional experiments may also involve individual gene knockouts (including complementation) for gene functional characterization. These follow-up studies using other *S. enterica* strains and food models will lead to a better understanding of the survival mechanisms, stress physiology, transmission, and epidemiology of this pathogen in different low-moisture foods.

## Data Availability Statement

All datasets presented in this study are included in the article/Supplementary Material.

## Author Contributions

YL, MM, and WZ designed the experiments. YL, PD, SP, WC, P-SP, OJ, and MM performed the experiments and analyzed the data. YL wrote the manuscript draft. YL, JS, YH, MT, OJ, HF, MM, SP, and WZ edited and approved the manuscript. WZ and MM secured the funding. All authors contributed to the article and approved the submitted version.

## Conflict of Interest

The authors declare that the research was conducted in the absence of any commercial or financial relationships that could be construed as a potential conflict of interest.
