# Supplementary material for: Mechanisms of Salmonella Attachment and Survival on In-Shell Black Peppercorns, Almonds, and Hazelnuts
Source: Front Microbiol. 2020 Oct 23;11:582202. doi: 10.3389/fmicb.2020.582202 (PMC7644838; doi:10.3389/fmicb.2020.582202)
Supplement: Supplementary file 1 [file Data_Sheet_1.docx]

**SUPPLEMENTARY MATERIAL**

**Supplementary Table 1. Equilibrium relative humidity according to selected saturated salt solutions.***

| Saturated salts | Temperature (°C) | Relative humidity (%) |
| --- | --- | --- |
| Lithium Iodide | 5 | 21.68 ± 0.30 |
| Magnesium Nitrate | 5 | 58.86 ± 0.43 |
| Potassium Acetate | 25 | 22.51 ± 0.32 |
| Sodium Bromide | 25 | 57.57 ± 0.40 |

*, indicates an evaluated saturated relative humidity vs. temperature for saturated salt solution was previously described (Greenspan, 1977).

**Supplementary Table 2. EZ-Tn5 primer pairs.***

| Name | Sequences | Functions |
| --- | --- | --- |
| T7 | CTGTCTCTTATACACATCTCAACCCTG-N18- AGGCCGGTCTCCCTATAGTGAGTCGTATTAATTTCGATAAG | Library construction |
| Kan2 | CTGTCTCTTATACACATCTCAACCATCA-N18- TCGATGAATTGTGTCTCAAAATCT CTGA | Library construction |
| 1^st^_Tn5_left | GTCTCTTATACACATCTCAACCATC | Library screening |
| 1^st^_Tn5_right | AATTGGTTGTAACACTGGCAGAGCATTA | Library screening |
| 2^nd^_Tn5_left | AATGATACGGCGACCACCGAGGCCATTAATACGACTCACTATAGGGAGACCGGCC | Library screening |
| 2^nd^_Tn5_right | CAAGCAGAAGACGGCATACGAGAT-N8-CCCGTCCCACCGTCTCTTATACAC ATCTCAACC | Library screening |
| Tn5_right_seq | GGCCATTAATACGACTCACTATAGGGAGACCGGCCT | Library screening |
| Tn5_index_seq | GGTTGAGATGTGTATAAGAGACGGTGGGACGGG | Library screening |

*, indicates Tn5 library construction was conducted as described previously (de Moraes et al., 2017; de Moraes et al., 2018; Jayeola et al., 2020).

**Supplementary Table 3. Log reduction of *S. enterica* on shell surface over 14-day storage at 25^o^C and 60% relative humidity.**

| Food sample | Media | Day | Mean ± SD log CFU/g | | | | |
| --- | --- | --- | --- | --- | --- | --- | --- |
|  |  |  | *S.* Typhimurium | *S.* Montevideo | *S.* Braenderup | *S.* Mbandanka | *S.* Enteritidis |
| Black pepper | BHI | 0 | 7.67 ± 0.17 ^Aa^ | 7.85 ± 0.18 ^ABa^ | 8.09 ± 0.04 ^Ab^ | 8.17 ± 0.11 ^Ab^ | 6.55 ± 0.13 ^Ac^ |
|  |  | 1 | 7.55 ± 0.32 ^ABa^ | 7.75 ± 0.03 ^ADa^ | 7.91 ± 0.09 ^BCb^ | 8.03 ± 0.11 ^Bb^ | 5.70 ± 0.08 ^Bc^ |
|  |  | 3 | 7.40 ± 0.19 ^Ba^ | 7.60 ± 0.08 ^Cab^ | 7.70 ± 0.14 ^CDb^ | 7.94 ± 0.05 ^Dc^ | 5.15 ± 0.24 ^Dd^ |
|  |  | 7 | 7.37 ± 0.18 ^Bab^ | 7.43 ± 0.12 ^Ea^ | 7.49 ± 0.12 ^Eab^ | 7.70 ± 0.15 ^EFb^ | 4.69 ± 0.31 ^Fc^ |
|  |  | 14 | 7.08 ± 0.21 ^Da^ | 7.23 ± 0.22 ^Fab^ | 7.29 ± 0.12 ^Gb^ | 7.29 ± 0.17 ^Gab^ | 4.02 ± 0.21 ^Hc^ |
|  | XLD | 0 | 7.61 ± 0.19 ^Aa^ | 7.89 ± 0.17 ^Aab^ | 8.06 ± 0.03 ^Ab^ | 8.19 ± 0.12 ^Abc^ | 6.45 ± 0.07 ^Ad^ |
|  |  | 1 | 7.49 ± 0.32 ^ABa^ | 7.70 ± 0.04 ^BCa^ | 7.83 ± 0.05 ^Db^ | 7.94 ± 0.07 ^Cb^ | 5.53 ± 0.12 ^Cc^ |
|  |  | 3 | 7.25 ± 0.18 ^Ca^ | 7.57 ± 0.14 ^CDb^ | 7.65 ± 0.15 ^Ebc^ | 7.78 ± 0.04 ^DEc^ | 4.95 ± 0.26 ^Ed^ |
|  |  | 7 | 7.26 ± 0.22 ^Cab^ | 7.31 ± 0.08 ^FGa^ | 7.41 ± 0.06 ^Fab^ | 7.50 ± 0.17 ^Fb^ | 4.45 ± 0.31 ^Gc^ |
|  |  | 14 | 6.91 ± 0.29 ^Ea^ | 7.04 ± 0.28 ^Ga^ | 7.18 ± 0.13 ^Ha^ | 6.99 ± 0.20 ^Ha^ | 3.78 ± 0.25 ^Ib^ |
| Almond | BHI | 0 | 7.65 ± 0.20 ^Aa^ | 7.77 ± 0.18 ^Aa^ | 8.08 ± 0.14 ^Ab^ | 8.18 ± 0.15 ^Ab^ | 7.44 ± 0.26 ^Aa^ |
|  |  | 1 | 7.10 ± 0.25 ^BCa^ | 7.45 ± 0.10 ^Bb^ | 7.68 ± 0.24 ^Bbc^ | 7.87 ± 0.17 ^BDc^ | 6.63 ± 0.33 ^Bd^ |
|  |  | 3 | 7.07 ± 0.08 ^Ba^ | 7.27 ± 0.04 ^Cb^ | 7.53 ± 0.21 ^BCc^ | 7.69 ± 0.18 ^CDc^ | 6.05 ± 0.18 ^Cd^ |
|  |  | 7 | 6.78 ± 0.18 ^CDEa^ | 7.09 ± 0.17 ^Db^ | 7.30 ± 0.22 ^CDbc^ | 7.41 ± 0.27 ^CEc^ | 5.48 ± 0.34 ^Ed^ |
|  |  | 14 | 6.71 ± 0.11 ^Dab^ | 6.65 ± 0.36 ^Ea^ | 6.83 ± 0.41 ^Fab^ | 6.94 ± 0.41 ^Gb^ | 4.68 ± 0.36 ^Gc^ |
|  | XLD | 0 | 7.66 ± 0.19 ^Aa^ | 7.86 ± 0.26 ^Aab^ | 8.07 ± 0.15 ^ABb^ | 8.10 ± 0.12 ^ABb^ | 7.49 ± 0.23 ^Aa^ |
|  |  | 1 | 7.11 ± 0.30 ^BCa^ | 7.35 ± 0.17 ^Cab^ | 7.58 ± 0.24 ^CDb^ | 7.70 ± 0.18 ^CEbc^ | 6.61 ± 0.29 ^Bd^ |
|  |  | 3 | 7.01 ± 0.07 ^Ca^ | 7.10 ± 0.09 ^Da^ | 7.42 ± 0.18 ^DEb^ | 7.47 ± 0.18 ^EFb^ | 5.94 ± 0.16 ^Dc^ |
|  |  | 7 | 6.68 ± 0.27 ^CDEa^ | 6.90 ± 0.30 ^DEab^ | 7.16 ± 0.20 ^EFb^ | 7.14 ± 0.40 ^FGb^ | 5.40 ± 0.33 ^Fc^ |
|  |  | 14 | 6.57 ± 0.14 ^Eab^ | 6.42 ± 0.48 ^Fa^ | 6.73 ± 0.44 ^Gab^ | 6.70 ± 0.48 ^Hb^ | 4.50 ± 0.43 ^Hc^ |
| Hazelnut | BHI | 0 | 6.95 ± 0.20 ^Aa^ | 7.22 ± 0.10 ^Aab^ | 7.13 ± 0.18 ^Ac^ | 7.42 ± 0.04 ^Ab^ | 5.89 ± 0.28 ^Ad^ |
|  |  | 1 | 6.48 ± 0.21 ^Ba^ | 6.83 ± 0.21 ^Ba^ | 6.80 ± 0.37 ^Bb^ | 7.11 ± 0.13 ^Bab^ | 4.99 ± 0.33 ^Bc^ |
|  |  | 3 | 6.06 ± 0.14 ^Da^ | 6.50 ± 0.21 ^Cbc^ | 6.06 ± 0.45 ^Dc^ | 6.65 ± 0.21 ^Dab^ | 4.07 ± 0.17 ^Dd^ |
|  |  | 7 | 5.46 ± 0.34 ^Fa^ | 5.70 ± 0.54 ^Ea^ | 5.58 ± 0.48 ^Fa^ | 5.67 ± 0.33 ^Ea^ | 3.43 ± 0.48 ^EFb^ |
|  |  | 14 | 4.80 ± 0.42 ^Ha^ | 4.88 ± 0.38 ^Ga^ | 4.58 ± 0.89 ^Ga^ | 4.63 ± 0.22 ^Ga^ | 2.34 ± 0.47 ^Gb^ |
|  | XLD | 0 | 6.93 ± 0.22 ^Aa^ | 7.17 ± 0.13 ^Aa^ | 7.10 ± 0.19 ^Ab^ | 7.42 ± 0.07 ^Aa^ | 5.99 ± 0.39 ^Ac^ |
|  |  | 1 | 6.34 ± 0.23 ^Ca^ | 6.63 ± 0.24 ^Ca^ | 6.63 ± 0.36 ^Cb^ | 6.97 ± 0.14 ^Cab^ | 4.84 ± 0.42 ^Cc^ |
|  |  | 3 | 5.95 ± 0.18 ^Ea^ | 6.31 ± 0.24 ^Dab^ | 5.82 ± 0.57 ^Eb^ | 6.44 ± 0.17 ^EFa^ | 3.98 ± 0.20 ^Ec^ |
|  |  | 7 | 5.28 ± 0.31 ^Ga^ | 5.59 ± 0.52 ^Fa^ | 5.44 ± 0.54 ^Fa^ | 5.60 ± 0.41 ^Fa^ | 3.40 ± 0.41 ^Fb^ |
|  |  | 14 | 4.61 ± 0.47 ^Ia^ | 4.62 ± 0.41 ^Ha^ | 4.38 ± 0.89 ^Ha^ | 4.38 ± 0.18 ^Ha^ | 2.03 ± 0.58 ^Gb^ |

All data are based on three independent biological replicates. Different capital letters indicate significant difference (*p*<0.05) among the population of the same strain on the same food surface at different sampling time points (columns). Different lowercase letters indicate significant difference (*p*<0.05) among the population of different *S. enterica* strains at the same sampling time point (rows).

**Supplementary Table 4. List of *S.* Enteritidis mutants selected against on surface of almonds.**

| Gene code | Annotation | *p*-value  D0 vs D1 | *p*-value  D0 vs D3 | *p*-value  D0 vs D7 | *p*-value  D0 vs D14 | *p*-value  D1 vs D3 | *p*-value  D1 vs D7 | *p*-value  D1 vs D14 |
| --- | --- | --- | --- | --- | --- | --- | --- | --- |
| *dnaK* | molecular chaperone | 2.1E-05 | 9.7E-01 | 1.0E-02 | 8.3E-03 | 1.8E-06 | 3.6E-01 | 4.0E-01 |
| *dapB* | dihydrodipicolinate reductase | 5.3E-05 | 5.3E-05 | 5.3E-05 | 5.3E-05 | 1.0E+00 | 1.0E+00 | 1.0E+00 |
| *fruR* | fructose repressor; DNA-binding transcriptional regulator | 1.0E+00 | 3.3E-02 | 1.5E-02 | 1.3E-06 | 8.7E-02 | 4.2E-02 | 5.6E-06 |
| *thiI* | thiamine biosynthesis protein | 1.0E+00 | 9.6E-01 | 3.7E-05 | 3.3E-03 | 1.0E+00 | 1.1E-04 | 7.9E-03 |
| *cyoD* | cytochrome o ubiquinol oxidase subunit IV | 9.0E-01 | 8.5E-02 | 1.7E-04 | 2.7E-06 | 4.4E-01 | 3.3E-03 | 7.3E-05 |
| *cyoB* | cytochrome o ubiquinol oxidase subunit I | 1.1E-01 | 7.9E-03 | 1.2E-04 | 4.2E-06 | 8.5E-01 | 1.6E-01 | 1.8E-02 |
| *cyoA* | cytochrome o ubiquinol oxidase subunit II | 6.1E-01 | 6.1E-02 | 3.6E-03 | 5.1E-05 | 7.0E-01 | 1.6E-01 | 6.2E-03 |
| *hupB* | transcriptional regulator; DNA-binding protein | 1.0E+00 | 9.7E-01 | 6.7E-02 | 6.1E-07 | 9.7E-01 | 6.5E-02 | 5.8E-07 |
| *hha* | hemolysin expression-modulating protein | 5.5E-01 | 5.7E-09 | 6.0E-07 | 1.4E-06 | 2.1E-06 | 1.8E-04 | 3.9E-04 |
| *acrB* | acridine efflux pump; multidrug efflux system AcrAB-TolC, inner-membrane proton/drug antiporter (RND type); acriflavin resistance protein B | 6.7E-01 | 8.7E-05 | 2.9E-09 | 1.1E-08 | 7.1E-03 | 5.3E-07 | 2.0E-06 |
| *acrA* | acridine efflux pump; multidrug efflux system AcrAB-TolC, membrane fusion component; acriflavin resistance protein A precursor | 6.2E-01 | 3.6E-05 | 1.5E-08 | 3.3E-08 | 4.5E-03 | 3.8E-06 | 8.0E-06 |
| *nagC* | N-acetylglucosamine operon transcriptional repressor; N-acetylglucosamine-6P-responsive transcriptional repressor | 1.0E+00 | 2.3E-01 | 3.7E-01 | 5.7E-03 | 1.1E-01 | 1.9E-01 | 1.7E-03 |
| *gltA* | type II citrate synthase | 9.8E-01 | 8.5E-07 | 2.5E-10 | 1.3E-09 | 6.4E-06 | 1.8E-09 | 1.0E-08 |
| *sdhA* | succinate dehydrogenase flavoprotein subunit | 7.6E-01 | 2.7E-01 | 1.0E+00 | 3.0E-03 | 1.8E-02 | 6.7E-01 | 5.4E-05 |
| *sucD* | succinyl-CoA synthetase subunit alpha | 8.9E-01 | 7.6E-01 | 9.8E-01 | 6.3E-05 | 2.3E-01 | 5.7E-01 | 2.1E-06 |
| *uvrB* | excinuclease ABC subunit B | 9.9E-01 | 7.8E-01 | 1.8E-01 | 2.8E-09 | 5.3E-01 | 7.4E-02 | 5.5E-10 |
| *lonH* | putative protease; lon protease homolog | 6.1E-01 | 9.9E-01 | 6.3E-03 | 1.5E-03 | 8.8E-01 | 2.2E-01 | 8.6E-02 |
| *ompA* | outer membrane protein A precursor | 5.8E-01 | 1.1E-06 | 6.8E-08 | 1.2E-05 | 2.4E-04 | 1.9E-05 | 2.1E-03 |
| *uvrY* | response regulator | 9.4E-01 | 7.9E-01 | 7.5E-03 | 1.8E-05 | 3.4E-01 | 5.8E-02 | 2.7E-04 |
| *uvrC* | excinuclease ABC subunit C | 8.1E-01 | 4.6E-01 | 1.1E-03 | 2.4E-11 | 9.8E-01 | 3.0E-02 | 3.3E-10 |
| *proQ* | putative solute/DNA competence effector; RNA chaperone; activator of ProP | 9.8E-01 | 9.8E-01 | 1.0E-05 | 2.3E-06 | 8.0E-01 | 7.3E-05 | 1.8E-05 |
| *cspC* | cold shock-like protein | 9.8E-01 | 5.2E-02 | 1.2E-01 | 1.4E-02 | 1.0E-02 | 3.6E-01 | 6.8E-02 |
| *fadR* | fatty acid metabolism regulator | 4.1E-01 | 1.4E-02 | 8.2E-06 | 2.3E-09 | 5.4E-01 | 3.6E-03 | 2.1E-06 |
| *topA* | DNA topoisomerase I; omega protein I | 7.2E-01 | 1.0E+00 | 1.2E-04 | 4.9E-03 | 7.9E-01 | 7.8E-03 | 1.3E-01 |
| *rnb* | exoribonuclease II involved in mRNA degradation | 1.4E-01 | 6.0E-01 | 3.3E-05 | 1.7E-10 | 8.8E-01 | 5.9E-02 | 1.6E-06 |
| *glgB2 [J]* | putative alpha amylase; malto-oligosyltrehalose trehalohydrolase | 7.8E-01 | 9.9E-01 | 1.5E-02 | 2.3E-04 | 9.6E-01 | 2.3E-01 | 9.4E-03 |
| *treY* | putative glycosyl hydrolase; malto-oligosyltrehalose synthase | 5.0E-01 | 9.7E-01 | 4.0E-02 | 4.1E-03 | 8.6E-01 | 7.0E-01 | 2.4E-01 |
| *glgX_1* | putative glycosyl hydrolase; limit dextrin alpha-1,6-maltotetraose-hydrolase; putative glycogen debranching protein | 1.9E-01 | 7.9E-01 | 7.1E-03 | 2.3E-06 | 8.1E-01 | 6.9E-01 | 5.4E-03 |
| *ydiJ* | putative oxidase; conserved hypothetical FAD-binding protein | 7.7E-01 | 1.0E-01 | 1.2E-04 | 5.9E-04 | 6.6E-01 | 5.5E-03 | 2.1E-02 |
| *rfc* | O-antigen polymerase | 9.3E-01 | 6.1E-01 | 8.2E-05 | 9.9E-01 | 9.7E-01 | 1.2E-03 | 1.0E+00 |
| *astE* | succinylglutamate desuccinylase | 1.0E+00 | 1.1E-03 | 4.9E-04 | 4.7E-05 | 6.5E-04 | 2.8E-04 | 2.6E-05 |
| *astB* | succinylarginine dihydrolase | 4.4E-01 | 2.0E-02 | 3.7E-06 | 1.8E-06 | 5.9E-01 | 1.6E-03 | 8.6E-04 |
| *astD* | succinylglutamic semialdehyde dehydrogenase | 9.4E-01 | 3.8E-01 | 3.7E-02 | 2.0E-03 | 8.4E-01 | 2.1E-01 | 2.1E-02 |
| *astA* | arginine N-succinyltransferase | 1.6E-01 | 3.3E-02 | 7.1E-04 | 1.3E-07 | 9.6E-01 | 3.0E-01 | 6.2E-04 |
| *astC* | bifunctional succinylornithine transaminase/acetylornithine transaminase | 2.2E-01 | 1.1E-03 | 3.2E-06 | 1.5E-09 | 2.9E-01 | 5.5E-03 | 5.8E-06 |
| *rfbP* | undecaprenol-phosphate galactosephosphotransferase/O-antigen transferase | 3.8E-01 | 4.1E-06 | 5.0E-11 | 1.7E-02 | 2.3E-03 | 3.4E-08 | 6.2E-01 |
| *rfbK* | phosphomannomutase | 1.0E+00 | 7.7E-01 | 5.4E-04 | 9.9E-01 | 8.5E-01 | 9.2E-04 | 9.8E-01 |
| *rfbN* | rhamnosyl transferase; LPS side chain defect | 7.4E-01 | 1.0E-04 | 3.3E-09 | 4.7E-01 | 5.8E-03 | 3.9E-07 | 9.9E-01 |
| *rfbC* | dTDP-4,deoxyrhamnose 3,5 epimerase; dTDP-4-dehydrorhamnose 3,5-epimerase | 9.5E-01 | 5.6E-02 | 8.6E-07 | 8.3E-01 | 2.7E-01 | 1.4E-05 | 1.0E+00 |
| *rfbD* | dTDP-4-dehydrorhamnose reductase | 7.6E-01 | 1.4E-03 | 1.7E-07 | 2.7E-01 | 4.5E-02 | 1.6E-05 | 9.2E-01 |
| *rfbB* | dTDP-glucose 4,6 dehydratase; NAD(P) binding | 8.4E-01 | 1.0E+00 | 2.3E-03 | 9.8E-01 | 6.9E-01 | 4.5E-02 | 9.9E-01 |
| *yegW* | putative gntR-family regulatory protein | 2.9E-01 | 6.5E-01 | 3.7E-07 | 4.3E-05 | 9.7E-01 | 5.3E-04 | 2.5E-02 |
| *nuoL* | NADH dehydrogenase subunit L | 9.9E-01 | 7.6E-02 | 4.6E-02 | 3.9E-04 | 1.9E-01 | 1.2E-01 | 1.6E-03 |
| *nuoI* | NADH dehydrogenase I, I subunit | 1.0E+00 | 1.9E-08 | 2.5E-11 | 2.5E-11 | 1.2E-08 | 2.3E-11 | 2.3E-11 |
| *nuoE* | NADH dehydrogenase subunit E | 1.0E+00 | 7.0E-01 | 1.3E-02 | 1.1E-02 | 6.7E-01 | 1.1E-02 | 9.3E-03 |
| *purR [D]* | putative LacI family transcriptional regulator | 2.9E-01 | 7.6E-03 | 8.5E-05 | 4.0E-06 | 5.5E-01 | 4.2E-02 | 4.0E-03 |
| *vacJ* | Outer-membrane-phospholipid-binding lipoprotein | 6.7E-01 | 9.1E-02 | 1.1E-03 | 1.8E-03 | 7.4E-01 | 5.6E-02 | 8.0E-02 |
| *yfgC* | putative inner membrane or exported | 8.5E-01 | 7.3E-02 | 1.4E-03 | 1.6E-03 | 4.8E-01 | 2.8E-02 | 3.2E-02 |
| *ndk* | nucleoside diphosphate kinase | 9.9E-01 | 3.5E-02 | 1.3E-07 | 3.8E-08 | 9.5E-02 | 6.2E-07 | 1.9E-07 |
| *hscA* | chaperone protein | 3.6E-01 | 4.6E-01 | 1.3E-02 | 1.1E-05 | 1.0E+00 | 5.9E-01 | 6.1E-03 |
| *iscA* | putative iron-sulfur cluster assembly protein | 8.4E-01 | 6.2E-01 | 8.9E-03 | 3.4E-05 | 1.0E+00 | 1.3E-01 | 1.2E-03 |
| *smpA* | hypothetical small protein A | 3.8E-01 | 2.1E-04 | 2.2E-07 | 2.8E-07 | 5.2E-02 | 1.9E-04 | 2.3E-04 |
| *rpoS* | RNA polymerase sigma factor | 3.8E-10 | 8.2E-11 | 2.0E-11 | 2.0E-11 | 9.9E-01 | 5.6E-09 | 3.0E-11 |
| *barA* | hybrid sensory histidine kinase; signal transduction histidine-protein kinase | 9.9E-01 | 6.9E-01 | 1.6E-02 | 2.7E-03 | 4.0E-01 | 5.5E-02 | 1.1E-02 |
| *nlpI* | lipoprotein involved in cell division | 2.1E-01 | 2.7E-04 | 1.5E-07 | 7.6E-11 | 1.4E-01 | 4.4E-04 | 2.6E-07 |
| *pnp* | polyribonucleotide nucleotidyltransferase; polyadenylation bacterial, bacterial RNA-metabolizing Zn-dependent hydrolases | 1.0E+00 | 2.2E-01 | 4.3E-01 | 4.5E-03 | 2.7E-01 | 4.9E-01 | 6.1E-03 |
| *yrbB* | putative STAS domain; phospholipid ABC transporter-binding protein; possible anti-sigma factor antagonist | 1.8E-01 | 1.1E-01 | 2.4E-03 | 2.7E-03 | 1.0E+00 | 4.8E-01 | 5.0E-01 |
| *yrbC* | putative transport protein; putative ABC superfamily; possible exported protein | 8.7E-01 | 2.6E-01 | 2.6E-03 | 3.2E-03 | 8.2E-01 | 4.2E-02 | 5.0E-02 |
| *yrbD* | putative transport protein; putative ABC superfamily binding protein; possible exported protein | 5.6E-01 | 1.4E-01 | 4.6E-03 | 2.0E-03 | 9.1E-01 | 2.1E-01 | 1.2E-01 |
| *yrbE* | putative transport protein; putative ABC superfamily membrane protein; putative membrane protein | 6.0E-01 | 9.9E-02 | 1.1E-04 | 2.7E-05 | 8.2E-01 | 1.2E-02 | 3.8E-03 |
| *yrbF* | putative ABC transporter ATP-binding protein; ATP-binding subunit of a putative ABC toluene efflux transporter | 8.8E-01 | 3.1E-01 | 7.2E-03 | 1.5E-03 | 8.6E-01 | 8.9E-02 | 2.6E-02 |
| *yhbJ* | putative kinase; contains putative P-loop; RNase adapter protein | 3.5E-01 | 7.0E-05 | 1.6E-05 | 2.1E-07 | 2.7E-02 | 8.7E-03 | 2.2E-04 |
| *mdh* | malate dehydrogenase | 1.0E+00 | 3.6E-03 | 5.5E-06 | 1.6E-05 | 1.1E-02 | 2.3E-05 | 6.2E-05 |
| *ArcZ [P]* | Post-transcriptional regulator represses sdaC, STM3216 and tpx mRNAs | 1.0E+00 | 9.8E-01 | 1.4E-01 | 3.4E-03 | 1.0E+00 | 2.8E-01 | 1.0E-02 |
| *cafA* | ribonuclease G | 9.2E-01 | 8.5E-01 | 2.1E-02 | 6.2E-04 | 3.6E-01 | 1.6E-01 | 8.7E-03 |
| *glpR* | DNA-binding transcriptional repressor; glycerol-3-phosphate regulon repressor | 9.3E-01 | 1.9E-02 | 1.9E-03 | 6.9E-11 | 1.4E-01 | 2.1E-02 | 1.2E-09 |
| *glgA* | glycogen synthase; ADP-glucose transglucosylase | 5.5E-01 | 1.0E-02 | 8.9E-03 | 3.7E-07 | 3.4E-01 | 3.2E-01 | 1.1E-04 |
| *glgC* | glucose-1-phosphate adenylyltransferase | 7.1E-01 | 3.1E-02 | 3.8E-03 | 5.7E-07 | 4.2E-01 | 1.1E-01 | 6.5E-05 |
| *glgB* | 1,4-alpha-glucan (glycogen) branching enzyme, GH-13-type | 7.2E-01 | 3.1E-02 | 4.3E-02 | 8.3E-05 | 4.1E-01 | 4.9E-01 | 5.4E-03 |
| *rfaL* | O-antigen ligase; oligosaccharide repeat unit polymerase | 1.0E+00 | 7.0E-05 | 7.4E-08 | 3.5E-02 | 1.3E-04 | 1.5E-07 | 5.4E-02 |
| *rfaK* | putative hexose transferase; lipopolysaccharide 1,2-N-acetylglucosaminetransferase; lipopolysaccharide core biosynthetic protein | 9.8E-01 | 6.0E-01 | 2.1E-05 | 1.0E+00 | 2.9E-01 | 2.8E-06 | 9.7E-01 |
| *rfaJ* | lipopolysaccharide glucosyltransferase; UDP-D-glucose/galactosyl; lipopolysaccharide 1,2-glucosyltransferase | 9.6E-01 | 7.2E-02 | 2.9E-06 | 9.3E-01 | 2.8E-01 | 3.3E-05 | 9.6E-01 |
| *rbsK* | ribokinase | 6.4E-01 | 8.6E-01 | 2.0E-02 | 4.4E-05 | 1.0E+00 | 3.9E-01 | 4.7E-03 |
| *hslU* | ATP-dependent protease ATP-binding subunit | 7.3E-01 | 1.0E+00 | 8.0E-03 | 1.2E-02 | 7.6E-01 | 1.8E-01 | 2.3E-01 |
| *hupA* | transcriptional regulator HU subunit alpha; histone-like DNA-binding protein | 1.0E+00 | 4.8E-01 | 1.2E-02 | 6.3E-09 | 7.0E-01 | 4.3E-03 | 1.6E-09 |
| *pgi* | phosphoglucose isomerase | 2.2E-01 | 1.4E-02 | 3.0E-05 | 3.2E-04 | 7.8E-01 | 3.1E-02 | 1.5E-01 |
| *zur* | zinc uptake transcriptional repressor | 9.1E-01 | 9.8E-01 | 6.1E-04 | 2.7E-02 | 6.2E-01 | 9.4E-03 | 2.0E-01 |
| *uvrA* | excinuclease ABC subunit A | 8.6E-01 | 9.3E-01 | 9.6E-01 | 1.2E-07 | 3.9E-01 | 4.6E-01 | 2.5E-09 |
| *hfq* | RNA-binding protein; host factor-I protein(HF-I) | 6.2E-01 | 6.3E-03 | 4.2E-05 | 2.4E-07 | 2.1E-01 | 4.9E-03 | 4.9E-05 |
| *hflC* | FtsH protease regulator | 9.3E-01 | 5.4E-02 | 2.6E-01 | 1.5E-05 | 2.8E-01 | 7.2E-01 | 2.5E-04 |
| *fbp* | fructose-1,6-bisphosphatase | 2.4E-02 | 2.7E-01 | 2.8E-04 | 1.7E-05 | 8.2E-01 | 6.1E-01 | 1.9E-01 |
| *uxuR* | DNA-binding transcriptional repressor; uxu operon transcriptional regulator | 1.0E+00 | 7.2E-01 | 2.1E-05 | 1.8E-02 | 8.1E-01 | 3.7E-05 | 2.7E-02 |
| *yjjK* | putative ABC transporter ATP-binding protein; energy-dependent translational throttle protein | 5.9E-01 | 7.5E-01 | 1.1E-04 | 2.6E-03 | 1.0E+00 | 1.3E-02 | 1.3E-01 |
| *putA* | transcriptional repressor of PutA and PutP / proline dehydrogenase (Proline oxidase) / delta-1-pyrroline-5-carboxylate dehydrogenase | 2.5E-01 | 9.7E-01 | 3.4E-03 | 1.7E-02 | 5.8E-01 | 4.4E-01 | 7.8E-01 |
| *ygaU* | putative LysM domain | 9.7E-01 | 8.8E-01 | 1.1E-02 | 1.5E-02 | 1.0E+00 | 5.6E-02 | 7.4E-02 |
| *hutI* | imidazolonepropionase | 4.5E-01 | 9.6E-01 | 1.5E-03 | 8.9E-02 | 8.5E-01 | 1.5E-01 | 9.0E-01 |
| *pflB* | pyruvate formate acetyltransferase 1 | 6.1E-01 | 9.7E-01 | 1.2E-02 | 9.9E-01 | 2.6E-01 | 3.2E-01 | 8.5E-01 |
| *pspA* | phage shock protein A; suppressor of sigma54-dependent transcription | 8.2E-01 | 8.5E-01 | 1.0E-01 | 3.3E-04 | 1.0E+00 | 6.2E-01 | 1.0E-02 |
| *ydaA* | putative universal stress protein | 1.8E-01 | 2.2E-05 | 1.6E-08 | 1.2E-10 | 3.2E-02 | 8.0E-05 | 6.8E-07 |
| *CDS* | putative cytoplasmic protein | 1.0E+00 | 1.2E-01 | 2.4E-05 | 1.8E-02 | 6.9E-02 | 9.3E-06 | 8.6E-03 |
| *cfa* | cyclopropane fatty acyl phospholipid synthase | 1.0E+00 | 5.2E-01 | 2.3E-03 | 2.5E-02 | 4.3E-01 | 1.5E-03 | 1.7E-02 |
| *sppA* | protease 4; essential to maintain secretion of mature proteins across the membrane | 6.3E-01 | 9.1E-01 | 9.8E-04 | 1.5E-01 | 9.8E-01 | 5.8E-02 | 8.8E-01 |
| *nuoG* | NADH dehydrogenase subunit G | 7.8E-01 | 1.8E-01 | 5.9E-01 | 3.6E-02 | 1.1E-02 | 8.3E-02 | 1.2E-03 |
| *hisM* | histidine/lysine/arginine/ornithine transport protein; histidine transport system permease protein; ABC superfamily membrane protein | 4.7E-02 | 7.8E-01 | 2.3E-03 | 8.7E-01 | 4.6E-01 | 8.3E-01 | 3.5E-01 |
| *ygbE* | putative inner membrane protein | 7.9E-01 | 1.0E+00 | 3.3E-03 | 1.0E+00 | 5.9E-01 | 7.5E-02 | 8.5E-01 |
| *rfaI* | lipopolysaccharide 1,3-galactosyltransferase | 3.0E-01 | 9.9E-01 | 1.3E-02 | 9.6E-01 | 5.4E-01 | 6.6E-01 | 8.0E-02 |
| *ubiB_2* | FMN reductase; NAD(P)H-flavin reductase | 1.0E+00 | 1.5E-01 | 5.0E-01 | 1.1E-03 | 3.0E-01 | 7.3E-01 | 3.4E-03 |
| *hslV* | ATP-dependent protease peptidase subunit; heat shock protein | 7.9E-01 | 9.8E-01 | 3.5E-02 | 1.8E-02 | 4.7E-01 | 3.7E-01 | 2.5E-01 |
| *miaA* | tRNA delta(2)-isopentenylpyrophosphate transferase; IPP transferase; isopentenyltransferase | 9.7E-01 | 1.8E-01 | 1.6E-02 | 7.1E-03 | 5.1E-01 | 8.5E-02 | 4.4E-02 |
| *hflK* | FtsH protease regulator | 1.0E+00 | 3.4E-01 | 3.0E-01 | 4.7E-04 | 5.7E-01 | 5.2E-01 | 1.6E-03 |
| *ytfK* | putative cytoplasmic protein | 9.9E-01 | 9.8E-01 | 5.3E-01 | 8.0E-04 | 1.0E+00 | 8.0E-01 | 3.4E-03 |
| *trpR* | Trp operon repressor | 5.8E-01 | 6.1E-01 | 1.3E-01 | 3.5E-04 | 1.0E+00 | 8.9E-01 | 3.4E-02 |

Red labels indicate the output reads of the mapped mutants have a significant difference (*p*<0.05) compared to the input reads (day zero or day one).

**Supplementary Table 5. List of *S.* Enteritidis mutants selected for on surface of almonds.**

| Gene code | Annotation | *p*-value  D0 vs D1 | *p*-value  D0 vs D3 | *p*-value  D0 vs D7 | *p*-value  D0 vs D14 | *p*-value  D1 vs D3 | *p*-value  D1 vs D7 | *p*-value  D1 vs D14 |
| --- | --- | --- | --- | --- | --- | --- | --- | --- |
| *ftsL* | membrane bound cell division protein | 1.0E+00 | 5.7E-03 | 1.0E-03 | 1.2E-04 | 1.5E-02 | 3.0E-03 | 3.8E-04 |
| *ddl_2* | D-alanine--D-alanine ligase | 7.6E-01 | 3.8E-03 | 1.5E-04 | 5.3E-11 | 9.7E-02 | 7.6E-03 | 3.5E-09 |
| *clpA* | ATP-dependent Clp protease; ATP-binding specificity subunit of the ClpA-ClpP | 9.5E-01 | 2.0E-03 | 9.4E-01 | 8.1E-04 | 1.7E-02 | 1.0E+00 | 8.0E-03 |
| *minD* | cell division inhibitor; ATPase; septum site-determining protein | 5.5E-01 | 8.1E-01 | 3.1E-02 | 5.5E-05 | 9.9E-01 | 5.9E-01 | 8.7E-03 |
| *minC* | septum formation inhibitor; septum site-determining protein | 1.4E-01 | 4.0E-01 | 8.1E-03 | 3.8E-05 | 9.8E-01 | 7.9E-01 | 6.2E-02 |
| *osmB* | lipoprotein; osmotically inducible lipoprotein B | 9.1E-01 | 4.1E-01 | 1.0E+00 | 4.6E-05 | 9.0E-01 | 9.8E-01 | 9.5E-04 |
| *osmE* | DNA-binding transcriptional activator; osmotically-inducible lipoprotein E; activator of ntr-like gene protein | 1.5E-01 | 9.8E-01 | 1.1E-01 | 1.4E-05 | 4.0E-02 | 1.0E+00 | 3.0E-02 |
| *yehR* | putative lipoprotein | 1.7E-01 | 3.2E-02 | 1.0E-03 | 3.9E-05 | 9.5E-01 | 3.4E-01 | 5.0E-02 |
| *eutA* | reactivating factor for ethanolamine ammonia lyase; putative ethanolamine utilization protein | 8.6E-01 | 1.9E-02 | 4.2E-02 | 7.2E-05 | 1.9E-01 | 3.3E-01 | 2.0E-03 |
| *stpA* | DNA binding protein, nucleoid-associated | 6.0E-02 | 2.1E-02 | 2.7E-05 | 1.0E-05 | 9.9E-01 | 1.2E-01 | 6.6E-02 |
| *ygbD* | nitric oxide reductase; hydrogenase maturation protein; putative rubredoxin reductase | 7.0E-01 | 8.2E-02 | 1.6E-04 | 3.4E-05 | 6.8E-01 | 1.1E-02 | 2.9E-03 |
| *yohL* | putative cytoplasmic protein; transcriptional repressor | 6.4E-02 | 2.4E-04 | 2.2E-08 | 6.2E-11 | 3.6E-01 | 5.6E-04 | 1.7E-06 |
| *sufI* | repressor protein for FtsI; SufI protein precursor; putative periplasmic protein | 4.6E-01 | 3.8E-06 | 6.2E-05 | 7.2E-11 | 1.5E-03 | 1.5E-02 | 3.7E-08 |
| *yhbC* | clustered with transcription termination protein NusA; bacterial ribosome SSU maturation protein | 4.0E-01 | 3.4E-01 | 1.4E-04 | 1.8E-05 | 1.0E+00 | 3.5E-02 | 7.1E-03 |
| *yrbK* | putative exported protein; lipopolysaccharide export system protein | 4.9E-01 | 3.4E-02 | 3.8E-06 | 5.6E-06 | 6.6E-01 | 1.2E-03 | 1.7E-03 |
| *fum-1 [J]* | L(+)-tartrate dehydratase subunit beta | 9.2E-01 | 1.3E-01 | 4.0E-03 | 1.3E-03 | 5.1E-01 | 4.1E-02 | 1.5E-02 |
| *yhdA* | regulatory protein; RNase E specificity factor; putative lipoprotein | 4.4E-01 | 1.0E+00 | 6.3E-01 | 9.1E-05 | 3.4E-01 | 1.0E+00 | 2.1E-02 |
| *fusA* | translation elongation factor G; EF-G; | 2.9E-02 | 2.9E-03 | 4.0E-06 | 1.1E-05 | 9.3E-01 | 7.3E-02 | 1.3E-01 |
| *glgP* | glycogen phosphorylase | 1.0E+00 | 5.6E-03 | 9.6E-01 | 7.8E-05 | 5.2E-03 | 9.5E-01 | 7.0E-05 |
| *yhhL* | putative membrane protein | 1.0E+00 | 6.5E-02 | 2.9E-03 | 8.1E-07 | 1.2E-01 | 6.8E-03 | 2.3E-06 |
| *rfaC* | ADP-heptose: LPS heptosyl transferase I; lipopolysaccharide heptosyltransferase-1 | 1.0E-03 | 2.3E-04 | 1.2E-08 | 2.0E-11 | 9.9E-01 | 2.7E-02 | 4.5E-06 |
| *rfaG* | UDP-glucose:(heptosyl) LPS alpha1,3-glucosyltransferase; lipopolysaccharide core biosynthesis protein | 8.6E-02 | 1.0E-01 | 3.9E-07 | 2.9E-06 | 1.0E+00 | 4.0E-03 | 1.9E-02 |
| *dut* | deoxyuridine 5'-triphosphate nucleotidohydrolase | 1.4E-02 | 5.1E-02 | 6.7E-04 | 9.4E-04 | 9.9E-01 | 8.7E-01 | 9.1E-01 |
| *atpB* | F0F1 ATP synthase subunit A | 3.8E-01 | 3.9E-02 | 1.8E-05 | 1.1E-06 | 8.0E-01 | 8.0E-03 | 7.5E-04 |
| *wecF* | putative entero common antigen polymerase; probable 4-alpha-L-fucosyltransferase | 1.8E-01 | 5.1E-03 | 4.2E-07 | 1.6E-08 | 6.4E-01 | 1.5E-03 | 8.3E-05 |
| *ubiB* | putative ubiquinone biosynthesis protein | 3.5E-03 | 6.3E-02 | 1.6E-05 | 1.4E-03 | 8.4E-01 | 5.3E-01 | 1.0E+00 |
| *ftsX* | cell division protein; putative ABC transporter, membrane protein | 8.6E-02 | 1.0E+00 | 1.0E-01 | 1.6E-02 | 4.4E-02 | 1.0E+00 | 9.7E-01 |
| *ftsE* | cell division protein; putative ATP-binding protein of an ATP-binding cassette transporter | 3.6E-02 | 1.0E+00 | 9.5E-02 | 2.5E-03 | 3.7E-02 | 9.9E-01 | 8.9E-01 |
| *dsbA* | periplasmic protein disulfide isomerase I; thiol-disulfide interchange protein DabA precursor | 9.8E-01 | 9.9E-01 | 5.9E-01 | 7.6E-03 | 8.5E-01 | 8.8E-01 | 3.3E-02 |
| *ftsN* | essential cell division protein | 2.8E-03 | 3.1E-03 | 2.8E-05 | 8.9E-09 | 1.0E+00 | 6.7E-01 | 9.1E-03 |
| *dcuB* | anaerobic C4-dicarboxylate transporter | 6.9E-02 | 1.0E-03 | 4.7E-06 | 6.0E-06 | 5.9E-01 | 3.4E-02 | 4.1E-02 |
| *ecnA* | putative entericidin A precursor | 1.0E+00 | 3.3E-02 | 9.7E-03 | 3.3E-05 | 6.2E-02 | 2.0E-02 | 8.2E-05 |
| *treF* | cytoplasmic trehalase | 6.3E-01 | 1.4E-02 | 2.5E-01 | 1.1E-03 | 3.3E-01 | 9.6E-01 | 6.6E-02 |
| *glnD* | PII uridylyl-transferase | 1.0E+00 | 5.7E-01 | 1.0E+00 | 2.0E-03 | 5.2E-01 | 1.0E+00 | 1.5E-03 |
| *yebA* | hypothetical protein | 3.6E-01 | 9.7E-01 | 3.0E-01 | 3.1E-03 | 1.1E-01 | 1.0E+00 | 3.0E-01 |
| *pspC* | DNA-binding transcriptional activator; phage shock protein C | 1.0E+00 | 1.0E+00 | 9.9E-01 | 6.0E-03 | 1.0E+00 | 1.0E+00 | 1.0E-02 |
| *phoP* | DNA-binding transcriptional regulator; response regulator in two-component regulatory system with PhoQ; involved in magnesium starvation and stress | 9.2E-01 | 6.7E-01 | 9.6E-01 | 1.1E-03 | 9.9E-01 | 1.0E+00 | 1.4E-02 |
| *sspH2* | leucine-rich repeat-containing protein; phage protein | 5.7E-01 | 3.6E-01 | 3.0E-04 | 9.2E-05 | 1.0E+00 | 3.1E-02 | 1.2E-02 |
| *mglA* | galactose/methyl galaxtoside transporter ATP-binding protein | 9.9E-01 | 1.1E-01 | 3.4E-02 | 4.1E-03 | 3.0E-01 | 1.1E-01 | 1.8E-02 |
| *yfbQ* | alanine transaminase; broad specificity; family IV; | 8.1E-01 | 8.9E-01 | 1.8E-01 | 1.1E-04 | 1.0E+00 | 7.9E-01 | 4.4E-03 |
| *dedD* | putative septum-associated cell division protein | 9.1E-01 | 2.0E-01 | 3.4E-01 | 1.6E-05 | 6.8E-01 | 8.5E-01 | 3.6E-04 |
| *purM* | phosphoribosylaminoimidazole synthetase | 9.6E-01 | 1.9E-01 | 1.3E-01 | 7.5E-04 | 5.5E-01 | 4.4E-01 | 6.8E-03 |
| *yqjA* | Putative DedA family inner membrane protein | 1.0E+00 | 4.1E-02 | 1.2E-01 | 7.7E-03 | 5.8E-02 | 1.6E-01 | 1.1E-02 |
| *dacB* | D-alanyl-D-alanine carboxypeptidase/endopeptidase; penicillin binding protein 4; penicillin sensitive; | 1.0E+00 | 1.0E+00 | 1.0E+00 | 1.4E-02 | 1.0E+00 | 1.0E+00 | 8.6E-03 |
| *lpfE* | long polar fimbrial minor protein; PFE protein precursor | 4.8E-02 | 2.9E-02 | 7.3E-03 | 1.5E-03 | 1.0E+00 | 9.6E-01 | 7.6E-01 |
| *yiaF* | putative outer membrane lipoprotein | 1.0E+00 | 5.8E-02 | 1.0E+00 | 3.0E-02 | 8.2E-02 | 1.0E+00 | 4.4E-02 |
| *yibP* | hypothetical protein | 1.3E-02 | 1.0E+00 | 6.3E-02 | 2.2E-03 | 1.4E-02 | 9.7E-01 | 9.8E-01 |
| *sugR* | ATP binding protein | 1.0E+00 | 6.5E-02 | 1.5E-01 | 1.1E-02 | 9.4E-02 | 2.1E-01 | 1.8E-02 |
| *cytR* | DNA-binding transcriptional regulator | 8.5E-01 | 7.8E-03 | 9.7E-02 | 2.2E-03 | 1.1E-01 | 5.5E-01 | 4.2E-02 |
| *amiB* | N-acetylmuramoyl-l-alanine amidase II; N-acetylmuramoyl-L-alanine amidase AMIB; a murein hydrolase | 8.8E-02 | 1.0E+00 | 5.3E-01 | 7.5E-03 | 8.6E-02 | 8.5E-01 | 8.8E-01 |
| *ytfB* | putative cell envelope opacity-associated protein A | 1.0E+00 | 1.7E-02 | 3.2E-01 | 4.3E-03 | 1.4E-02 | 2.9E-01 | 3.5E-03 |
| *yjgA* | putative aspartate carbamoyltransferase catalytic subunit | 7.5E-01 | 3.8E-02 | 3.9E-03 | 5.4E-03 | 4.3E-01 | 9.9E-02 | 1.3E-01 |

Red labels indicate the output reads of the mapped mutants have a significant difference (*p*<0.05) compared to the input reads (day zero or day one).

**Supplementary Table 7. List of *Salmonella* mutants selection on surface of almonds and pistachios.**

| Gene code | *S.* Enteritidis selection on surface of almonds | *S.* Enteritidis selection on surface of pistachios | Other *Salmonella* serovars selection on surface of pistachio |
| --- | --- | --- | --- |
| *hupB* | Down | Down |  |
| *uvrB* | Down | Down |  |
| *uvrY* | Down | Down |  |
| *uvrC* | Down | Down | Down |
| *proQ* | Down | Down |  |
| *cspC* | Down | Down |  |
| *topA* | Down | Down |  |
| *rnb* | Down | Down |  |
| *glgB2 [J]* | Down | Down |  |
| *treY* | Down | Down |  |
| *glgX_1* | Down | Down |  |
| *rfbP* | Down | Down |  |
| *rfbK* | Down | Down |  |
| *rfbN* | Down | Down |  |
| *rfbC* | Down | Down |  |
| *rfbD* | Down | Down |  |
| *rfbB* | Down | Down | Down |
| *hscA* | Down | Down |  |
| *ygaU* | Down | Down |  |
| *rpoS* | Down | Down |  |
| *barA* | Down | Down |  |
| *nlpI* | Down | Down |  |
| *pnp* | Down | Down |  |
| *yhbJ* | Down | Down |  |
| *cafA* | Down | Down |  |
| *glgA* | Down | Down |  |
| *glgC* | Down | Down |  |
| *glgB* | Down | Down |  |
| *rfaL* | Down | Down |  |
| *rfaK* | Down | Down |  |
| *rfaJ* | Down | Down |  |
| *rbsK* | Down | Down |  |
| *hslU* | Down | Down |  |
| *hupA* | Down | Down |  |
| *pgi* | Down | Down |  |
| *uvrA* | Down | Down |  |
| *hfq* | Down | Down |  |
| *hflC* | Down | Down |  |
| *fbp* | Down | Down |  |
| *trpR* | Down | Down |  |
| *thiI* | Down | Tend down |  |
| *cyoD* | Down | Tend down |  |
| *hha* | Down | Tend down | Down |
| *pflB* | Down | Tend down |  |
| *ompA* | Down | Tend down |  |
| *ydaA* | Down | Tend down |  |
| *astD* | Down | Tend down |  |
| *yegW* | Down | Tend down | Down |
| *iscA* | Down | Tend down |  |
| *rfaI* | Down | Tend down | Down |
| *ubiB_2* | Down | Tend down | Down |
| *zur* | Down | Tend down | Down |
| *miaA* | Down | Tend down |  |
| *acrB* | Down | Unchanged |  |
| *acrA* | Down | Unchanged |  |
| *fadR* | Down | Unchanged |  |
| *astB* | Down | Unchanged |  |
| *astA* | Down | Unchanged |  |
| *CDS* | Down | Unchanged |  |
| *hisM* | Down | Unchanged |  |
| *vacJ* | Down | Unchanged |  |
| *yfgC* | Down | Unchanged |  |
| *ygbE* | Down | Unchanged |  |
| *yrbB* | Down | Unchanged |  |
| *yrbC* | Down | Unchanged |  |
| *yrbE* | Down | Unchanged |  |
| *hslV* | Down | Unchanged | Down |
| *hflK* | Down | Unchanged |  |
| *ytfK* | Down | Unchanged |  |
| *yjjK* | Down | Unchanged |  |
| *ArcZ [P]* | Down | ND | Down |
| *ftsL* | Up | Up | Up |
| *yhdA* | Up | Up | Up |
| *fusA* | Up | Up | Up |
| *yhhL* | Up | Up | Up |
| *treF* | Up | Up | Up |
| *yiaF* | Up | Up | Up |
| *yibP* | Up | Up | Up |
| *phoP* | Up | Tend up | Up |
| *glgP* | Up | Tend up | Up |
| *ftsN* | Up | Tend up | Up |
| *ytfB* | Up | Tend up | Up |
| *glnD* | Up | Unchanged |  |
| *clpA* | Up | Unchanged | Up |
| *yebA* | Up | Unchanged |  |
| *osmB* | Up | Unchanged |  |
| *osmE* | Up | Unchanged |  |
| *sspH2* | Up | Unchanged |  |
| *yehR* | Up | Unchanged |  |
| *mglA* | Up | Unchanged |  |
| *yfbQ* | Up | Unchanged |  |
| *eutA* | Up | Unchanged |  |
| *purM* | Up | Unchanged |  |
| *stpA* | Up | Unchanged | Up |
| *yohL* | Up | Unchanged |  |
| *sufI* | Up | Unchanged |  |
| *dacB* | Up | Unchanged |  |
| *fum-1 [J]* | Up | Unchanged |  |
| *ftsX* | Up | Unchanged |  |
| *ftsE* | Up | Unchanged |  |
| *lpfE* | Up | Unchanged |  |
| *rfaC* | Up | Unchanged |  |
| *sugR* | Up | Unchanged |  |
| *atpB* | Up | Unchanged |  |
| *ubiB* | Up | Unchanged |  |
| *dsbA* | Up | Unchanged | Up |

Down or up indicate the output reads show selection (*p*<0.05) in comparison to input reads (day zero or day one) for at least two time points. “Tend” down or up indicate the output reads show selection (*p*<0.05) in comparison to input reads (day zero or day one) at one time point. ND stands for not detected.
